# Catalase (KatA) Plays a Role in Protection against Anaerobic Nitric Oxide in *Pseudomonas aeruginosa*

**DOI:** 10.1371/journal.pone.0091813

**Published:** 2014-03-24

**Authors:** Shengchang Su, Warunya Panmanee, Jeffrey J. Wilson, Harry K. Mahtani, Qian Li, Bradley D. VanderWielen, Thomas M. Makris, Melanie Rogers, Cameron McDaniel, John D. Lipscomb, Randall T. Irvin, Michael J. Schurr, Jack R. Lancaster, Rhett A. Kovall, Daniel J. Hassett

**Affiliations:** 1 Department of Molecular Genetics, Biochemistry and Microbiology, University of Cincinnati, Cincinnati, Ohio, United States of America; 2 Departments of Anesthesiology, Cell, Developmental and Integrative Biology, and Environmental Health Sciences, University of Alabama at Birmingham, Birmingham, Alabama, United States of America; 3 Department of Biochemistry, Molecular Biology, and Biophysics, University of Minnesota, Minneapolis, Minnesota, United States of America; 4 Department of Medical Microbiology and Immunology, University of Alberta, Edmonton, Alberta, Canada; 5 Department of Microbiology, University of Colorado, Aurora, Colorado, United States of America; 6 Center for Free Radical Biology, University of Alabama at Birmingham, Birmingham, Alabama, United States of America; East Carolina University School of Medicine, United States of America

## Abstract

*Pseudomonas aeruginosa* (*PA*) is a common bacterial pathogen, responsible for a high incidence of nosocomial and respiratory infections. KatA is the major catalase of *PA* that detoxifies hydrogen peroxide (H_2_O_2_), a reactive oxygen intermediate generated during aerobic respiration. Paradoxically, *PA* displays elevated KatA activity under anaerobic growth conditions where the substrate of KatA, H_2_O_2_, is not produced. The aim of the present study is to elucidate the mechanism underlying this phenomenon and define the role of KatA in *PA* during anaerobiosis using genetic, biochemical and biophysical approaches. We demonstrated that anaerobic wild-type PAO1 cells yielded higher levels of *katA* transcription and expression than aerobic cells, whereas a nitrite reductase mutant Δ*nirS* produced ∼50% the KatA activity of PAO1, suggesting that a basal NO level was required for the increased KatA activity. We also found that transcription of the *katA* gene was controlled, in part, by the master anaerobic regulator, ANR. A Δ*katA* mutant and a mucoid *mucA22* Δ*katA* bacteria demonstrated increased sensitivity to acidified nitrite (an NO generator) in anaerobic planktonic and biofilm cultures. EPR spectra of anaerobic bacteria showed that levels of dinitrosyl iron complexes (DNIC), indicators of NO stress, were increased significantly in the Δ*katA* mutant, and dramatically in a Δ*norCB* mutant compared to basal levels of DNIC in PAO1 and Δ*nirS* mutant. Expression of KatA dramatically reduced the DNIC levels in Δ*norCB* mutant. We further revealed direct NO-KatA interactions *in vitro* using EPR, optical spectroscopy and X-ray crystallography. KatA has a 5-coordinate high spin ferric heme that binds NO without prior reduction of the heme iron (*K*_d_ ∼6 μM). Collectively, we conclude that KatA is expressed to protect *PA* against NO generated during anaerobic respiration. We proposed that such protective effects of KatA may involve buffering of free NO when potentially toxic concentrations of NO are approached.

## Introduction

*Pseudomonas aeruginosa* (*PA*) is a human pathogen that is currently ranked 5th in overall frequency of nosocomial infections in the United States [1]. Burn and cancer chemotherapy patients, chronic alcoholics, the immunocompromised (e.g., HIV infection), and patients suffering from chronic obstructive pulmonary disease (COPD) and cystic fibrosis (CF) lung disease are particularly prone to highly problematic or potentially lethal infections by this organism. During airway infection of both COPD [2] and CF patients [3], neutrophil recruitment to anaerobic infection sites can be nearly 100-fold higher [4] than normal accumulation levels observed during early aerobic phase of airway infection with both lacto- and myeloperoxidases being detected at high levels in sputum from patients with these infections. These innate immune cells generate antimicrobial hydrogen peroxide (H_2_O_2_) during the respiratory burst [5]. In fact, within the confines of the neutrophil phagolysosome, H_2_O_2_ concentrations have been estimated to be in the micromolar range [6], a concentration that is known to easily kill free-swimming planktonic cells, but not highly refractory *PA* biofilms [7], [8]. *PA* bacterial defense against H_2_O_2_ is mediated by three catalases (KatA, KatB, and KatC), and four alkyl hydroperoxide reductases (AhpA, AhpB, AhpCF, and Ohr), some of which have peroxidase and/or catalase activity. KatA is the major catalase in *PA* [9], [10]. The transcription of *katA* is reported to be regulated in part by the intercellular signaling system called quorum sensing [7], iron levels [11], [12], OxyR (a major H_2_O_2_ transcriptional activator) [13], IscR (an iron sulfur cluster assembly regulator) [13], [14] and the master anaerobic regulator ANR, a [4Fe-4S]^2+^ cluster protein [15], [16]. KatA is also a very stable enzyme, being highly resistant to a variety of proteases [11], [17] and is a recognized virulence factor in a murine infection model [18].

*PA* has a vast repertoire of enzymes and regulatory systems governing its response to H_2_O_2_, and it is not surprising that the organism tolerates it at millimolar concentrations. However, the organism is far more resistant to H_2_O_2_ when cultured in surface-attached communities known as biofilms. In fact, biofilm organisms are nearly 1000-fold more resistant than their planktonic counterparts to H_2_O_2_ [19], [20]. We have previously shown that KatA is critical for optimal resistance of *PA* grown in aerobic biofilm culture [21]. Recently, however, our laboratory has shown that catalase activity is higher in anaerobic *PA* cultures [19], despite the fact that its primary substrate, H_2_O_2_, is only produced metabolically via aerobic respiration or by the respiratory burst of phagocytes during infection, suggesting that KatA possesses functions beyond the removal of H_2_O_2_. Additionally, transcriptomic and proteomic studies by our laboratory have indicated that KatA expression was higher during anaerobic conditions relative to that observed under aerobic conditions [15]. Trunk et al., [16] have also suggested that *katA* transcription is, in part, dependent upon the [4Fe-4S]^2+^ cluster master anaerobic regulator, ANR (**A**naerobic **N**itrate **R**egulator) [22]. Thus, we hypothesized that KatA must provide a previously unrecognized anaerobic function in normal *PA* metabolism. Data from ours and other research groups suggests that chronically infected CF and COPD airway mucus progressively favors anaerobic metabolism by the pathogen [23]–[28].

One potential impediment to anaerobic growth of *PA* is the production of NO as an intermediate of anaerobic respiration (for review, see [29], [30]). The main defense against NO resides in NO reductase (NOR, [31]). NO can disrupt many aspects of the cellular physiology, but the reaction of NO with iron containing proteins (especially Fe-S cluster proteins [32] to produce a range of dinitrosyl iron complexes known collectively as DNICs [33], [34] is particularly disruptive. For example, Ren et al. [32] have shown that the Fe-S cluster of the *E. coli* dihydroxyacid dehydratase (IlvD) is inactivated by anaerobic NO, resulting in the formation of a stable IlvD-DNIC and other protein-bound DNICs. IlvD and other Fe-S containing proteins are part of the cellular “chelatable iron pool” (CIP, only 0.2–3% of total cellular iron and thus readily inactivated by low concentrations of NO [35]), and have been shown to be the major protein source of DNIC when microorganisms are under anaerobic NO stress [36]. NO can also react with phagocyte- or enzymatically (e.g., xanthine oxidase)-generated superoxide (O_2_^. −^) under aerobic conditions to generate the reactive, toxic oxidant, peroxynitrite (ONOO^−^). NO also participates in the anaerobic activation of DNR (**d**issimilatory **n**itrate respiration **r**egulator, [37]), a second-tier regulator that is controlled by ANR [38]. Thus, the production of NO via anaerobic respiration must be tightly regulated for normal cell function, due to its potentially harmful effects if either over-produced and/or not detoxified. For example, the negative effects of metabolic NO overproduction was manifested by an apparent NO-mediated “suicide” of organisms lacking the RhlR quorum sensing regulator in anaerobic biofilms [28]. Similarly, we have also shown that clinical mucoid, *mucA22* mutant bacteria are killed by NO generated by anaerobic reduction of exogenous acidified sodium nitrite (A-NO_2_^−^). Low pH (pH 6.4–6.5) is common in the airway mucus lining the COPD (e.g., long-term smoking, pH 5.8–6.3, [39]) and in CF airways [40].

The major anaerobic enzyme that minimizes the potentially toxic effects of NO is NOR [31], [41],[42]. Yoon et al., [42] showed that anaerobic *PA* lacking NOR accumulates nearly 13.6 μM NO. In response, the organism has a “circuit breaker”-like mechanism where NO inactivates the [4Fe-4S]^2+^ cluster of ANR, likely via formation of ANR-DNIC, to shut down endogenous production of NIR-dependent NO. This significantly reduces the intracellular NO concentration, affording cell survival, albeit at the cost of very slow growth.

In this study, we have explored the metabolic function underlying the increase in anaerobic versus aerobic catalase activity (specifically KatA) in *PA*. Increased anaerobic KatA activity was dependent upon normal metabolic NO production mediated by NIR activity and the global anaerobic regulator, ANR. However, KatA was observed to have additional roles namely: (i) found to be important for anaerobic viability of *PA* when exposed to acidified nitrite (A-NO_2_^−^) for both planktonic and biofilm associated cells, (ii) binds NO stoichiometrically, (iii) reduces formation of anaerobic DNICs, and (iv) is necessary for optimal growth upon a shift from anaerobic to aerobic conditions.

## Materials and Methods

### Bacterial strains, plasmids and planktonic growth conditions

All bacteria and plasmids used in this study are listed in **Table 1**. Bacteria were grown in either Luria-Bertani broth (LB) (tryptone, 10 g; yeast extract, 5 g; NaCl, 5 g for one liter of broth) or LB supplemented with 50 mM KNO_3_ (LBN). When specified, LB or LBN were buffered with 50 mM potassium phosphate to achieve acidic pH 6.5. Aerobic cultures were grown at 37°C with shaking at 200 rpm. Anaerobic cultures were grown in a duel-port, Coy Systems anaerobic chamber at 37°C in an atmosphere of 85% N_2_-10% CO_2_-5% H_2_. Media were solidified with 1.5% Bacto-agar. Frozen bacterial stocks were stored at −80°C in a 1∶1 mixture of 30% glycerol and aerobically grown, stationary phase bacteria.

**Table 1:** Bacterial strains, plasmids and oligonucleotides used in this study.

Strain, plasmid or oligonucleotide	Description (relevant genotype or phenotype) or sequence (5′ to 3′)	Source, reference, or RE site
*E. coli*		
DH5α	F^−^ Φ80d*lacZΔM15 endA1 recA1 hsdR17(r_K_* ^−^ *m_K_* ^−^ *) supE44 thi-1 gyrA96* Δ*(lacZYA-argF)*U169	Invitrogen
S17-1	Pro− Res− Mod+ *recA*; integrated RP4-Tet: : Mu-Kan:: Tn*7*, Mob+	[85]
BL21(DE3)(pLysS)	Strain used for T*7* promoter-based expression system	Novagen
*P. aeruginosa*		
PAO1	Wild-type, prototroph	[86]
Δ*katA*	Δ*katA*::Gm	[10]
Δ*rhlR*	Δ*rhlR*::Gm	[42]
Δ*katA*Δ*rhlR*	Δ*katA*::Tc, Δ*rhlR*::Gm	This study
Δ*nirS*	Δ*nirS*, unmarked	This study
Δ*katA*Δ*nirS*	Δ*nirS*::Gm, Δ*katA*::Tc	This study
Δ*norCB*	Δ*norCB*::Gm	[42]
Δ*katA*Δ*norCB*	Δ*katA*::Tc, Δ*norCB*::Gm	This study
Δ*katA*Δ*bfrA*	Δ*katA*Δ*bfrA*::Gm	[10]
Δ*katA*::*katA*	*katA* driven by its native promoter was integrated at chromosomal *attB* site of *katA* mutant	This study
Δ*katA*(pHERD*katA*)	Complemented *katA* mutant by plasmid-borne *katA*	This study
Plasmids		
pUCGM	Source for Gm^r^ cassette, Ap^R^, Gm^R^	[45]
pJFM18	*katA* cloned between *Nde*I and *Eag*I sites of pET23a, Ap^R^	[42]
pQF50	Broad-host-range transcriptional fusion vector with a promoterless *lacZ*, Ap^R^	[87]
mini-CTX1	Integration proficient plasmid for *P. aeruginosa*, Tc^R^	[47]
pEX100T-KS	*Pseudomonas* gene replacement suicide vector with modified multiple cloning site, *sacB*, *oriT*, Cb^R^	[46]
pQF50p*katA*	300-bp *katA* promoter cloned into *Sal*I/*Bam*HI sites of pQF50, Ap^R^	This study
mini-CTX1-*katA*	A 1.7 kb fragment containing *katA* gene and its own promoter region cloned into the vector pmini-CTX1, Tc^R^.	This study
pEXΔ*nirS*::FRT-Gm	A 2.3 kb fragment containing flanking sequences of *nirS* and FRT-Gm^R^-FRT cassette cloned into *Bam*HI/*Sal*I sites of pEX100T-KS, Cb^R^, Gm^R^	This study
pEXΔ*norCB*::Gm	A 3kb fragment containing flanking sequences of *norCB* and Gm^R^ cassette cloned into pEX100T-KS, Cb^R^, Gm^R^	[42]
pHERD20T	an *E. coli*-*Pseudomonas* shuttle vector with an arabinose-inducible P_BAD_ promoter, Ap^R^	[48]
pHERD*katA*	A 1,449 bp *katA* gene cloned into *Eco*RI/*Hind*III sites of pHERD20T, Ap^R^	This study
pHERD*nirS*	A 1,707 bp *nirS* gene cloned into *Nco*I/*Hind*III sites of pHERD20T, Ap^R^	This study
pHERD*anr*	A 735 bp *anr* gene cloned into *Kpn*I/*Hind*III sites of pHERD20T, Ap^R^	This study
Oligonucleotides		
p*katA*/XhSal5′	ACGCTCGAGTCGACGTAGAAGCTGCCGAAT	*Sal*I
p*katA*/Bam3′	CGGGATCCGGTCTTCTCTTCCATTTACTC	*Bam*HI
p*katA*/Bam5′	CGGGATCCGTAGAAGCTGCCGAATAAGGC	*Bam*HI
*katA*/Eco3′	GGAATTCTCAGTCCAGCTTCAGGCC	*Eco*RI
*katA*/Eco5′	GGAATTCGATGGAAGAGAAGACCCGC	*Eco*RI
*katA*/Hind3′	CCCAAGCTTCAGTCCAGCTTCAGGCC	*Hind*III
*nirS*/Nco5′	ATATCCATGGCATTTGGCAAGCCACTG	*Nco*I
*nirS*/Hind3′	CCCAAGCTTCAGTACACGTCGTGCTG	*Hind*III
*anr/*Kpn5′	CAAGGTACCAATGGCCGAAACCATCAAG	*Kpn*I
*anr/*Hind3′	CCCAAGCTTCAGCCTTCCAGCTGGCC	*Hind*III

Ap^R^, ampicillin resistant; Cb^R^, carbenicillin resistant; Tc^R^, tetracycline resistant; Gm^R^, gentamycin resistant.

### DNA manipulations

Genomic DNA isolation, PCR, restriction enzyme digestion, ligation, cloning and DNA electrophoresis were done according to standard techniques [43]. All oligonucleotide primers were synthesized by Integrated DNA Technologies (IDT). PCR was performed using either Choice *Taq* Mastermix (Denville Scientific, Inc. USA) or *Pfu* DNA polymerase (Strategene, USA). Plasmid isolation was performed using QIAprep Spin miniprep kits (QIAGEN) as recommended by the manufacturer. DNA fragments were purified using either a QIAquick PCR purification kit (QIAGEN) or a QIAquick gel extraction kit (QIAGEN). All cloned inserts were confirmed by automated DNA sequencing performed at the DNA Core Facility of the Cincinnati Children’s Hospital Medical Center. Plasmids were introduced into *E. coli* by CaCl_2_-mediated transformation and into *P. aeruginosa* by either electroporation or S17-1-mediated conjugation [44].

### Construction of *P. aeruginosa* mutants

The strategy for insertional inactivation of some of the genes listed in **Table 1** was facilitated by gene disruption with an 850-bp gentamicin resistance (Gm^R^) cassette from pUCGM [45], and the gene replacement vector pEX100T [46], the latter of which allowed for selection of double-crossover events within putative recombinants cultured on agar containing 5% sucrose. All mutants were confirmed by PCR analysis.

### DNA cloning and construction of recombinant plasmids and strains

A 300-bp promoter region of the *katA* gene of *P. aeruginosa* PAO1 was amplified by PCR with primer pair p*katA*/XhSal5′ and p*kat*A/Bm3′. The amplified promoter p*katA* was digested with *Sal*I and *Bam*HI, and cloned within the *Sal*I and *Bam*HI sites of pQF50, creating *pkatA*-*lacZ* transcriptional reporter plasmid pQF50p*katA*. A 1.7 kb fragment containing *katA* gene and its upstream promoter region was amplified by PCR with primers p*katA*/Bam5′ and *katA*/Eco3′, restricted with *Bam*HI and *Eco*R1, and cloned into the vector mini-CTX1[47]. The resultant plasmid mini-CTX1-*katA* was then used to introduce a wild-type copy of *katA* at the chromosomal *attB1* site of Δ*katA* mutant, generating the complementation strain Δ*katA*::*katA*. Open reading frames (ORF) of *katA*, *nirS*, and *anr* were amplified by PCR with primers *katA*/Eco5′ and *katA*/Hind3′, *nirS*/Nco5′ and *nirS*/Hind3′, and *anr*/Kpn5′ and *anr*/Hind3′, and cloned into vector pHERD20T [48], yielding pHERD*katA*, pHERD*nirS*, and pHERD*anr*, respectively.

### Enzymatic assays and Western blot analysis

Catalase activity was measured spectrophotometrically by following the decomposition of 19.5 mM H_2_O_2_ in potassium phosphate buffer, pH 7.0 at 240 nm [10], [49]. One unit of activity was defined as that which decomposed 1 μmol of H_2_O_2_ min^−1^ mg^−1^ protein. Overnight bacterial cultures (1.5 ml) under either aerobic or anaerobic conditions were harvested by centrifugation. Cell pellet was then suspended in 100 μl of PBS and disrupted by sonication. The suspension was clarified by centrifugation and total soluble proteins were used for catalase activity assay. β-galactosidase assays were performed as described by Miller [50] with slight modifications. Briefly, a 400 μl of bacterial culture at an OD_600_ of 1.0 was transferred to a tube containing 600 μl of Z-buffer, 20 μl of 0.1% SDS and 40 μl of chloroform, vortexed for 10 s, and incubated for 5 min at 28°C. An amount of 200 μl of orthonitrophenyl galactoside (4 mg/ml in 0.1 M phosphate buffer, pH7.0) was added and the reaction was allowed to proceed until yellow color developed. The reaction was stopped by the addition of 500 μl of 1 M Na_2_CO_3_. Samples were centrifuged to remove cell debris and chloroform, and the supernatant was measured at 420 nm. Aconitase was assayed by the production of *cis*-aconitate (3.6 mM^−1^cm^−1^at 240 nm) as described previously [51]. Protein concentration was estimated by the method of Bradford (4) using bovine serum albumin fraction V (Sigma) as standard. Cell extracts were subjected to 10% SDS-PAGE at 100 V for 1.5 hr. Separated proteins were then examined with coomassie blue staining or electro-blotted to PVDF membranes (Amersham, USA) using the Trans-Blot system (Bio-Rad, USA) at 120 mA for 2 hr. Membranes were washed and blocked in 5% dried skimmed milk in PBS-Tris-HCl buffer, pH 7.5. Then, the membranes were incubated with primary antibody (10^−4^ dilution) against KatA of *Streptomyces coelicolor*, known to cross-react with *PA* KatA [11] for 1 hr. Excess antibody was removed by three washings with PBS-T. After incubation for 1 hr in PBS-T containing the secondary antibody (10^−3^ dilution of peroxidase-conjugated, goat anti-mouse IgG), the membrane was washed three times with PBS-T. Immunodetection was performed using the ECL select Western blotting detection system according to the manufacturer’s instruction (Amersham, USA). The exposed X-ray film was recorded by scanning, and the relative density of reactive catalase bands was analyzed using the NIH ImageJ 1.47 g software.

### Acidified NO_2_^−^ sensitivity measurements

(i) Overnight cultures of *P. aeruginosa* wild-type PAO1, Δ*katA* mutant, Δ*katA*::*katA*, FRD1, and FRD1Δ*katA* were 1∶100 diluted into either LB broth (pH 6.5) or LBN broth (pH6.5) supplemented with varying concentrations of NaNO_2_ (0, 5, 10, 15, 20, 25 and 30 mM) and grown aerobically for 24 hrs, or anaerobically for 48 hrs. 5 μl of cells from each culture were spotted onto LB agar plates and incubated aerobically for 24 hrs at 37°C. The plates were then scanned for figure presentation. (ii) All strains above were also cultured anaerobically for 72 hrs in LBN broth (pH 6.5) supplemented with either 0, 20, and 25 mM NaNO_2_ for PAO1, Δ*katA* mutant and Δ*katA*::*katA*, or 0, and 5 mM NaNO_2_ for FRD1 and FRD1Δ*katA*, respectively. Cultures were taken daily, and serial cell dilutions were spotted onto LB broth agar plates. Surviving bacteria were enumerated after a 24 hr incubation at 37°C.

### Sensitivity of anaerobic biofilm to acidified NO_2_^−^

*P. aeruginosa* wild-type PAO1, D*katA* mutant, and D*katA*::*katA* strains were grown aerobically in LB broth to stationary phase followed by a 1∶100 dilution into 3 ml of LBN in confocal “friendly” glass bottomed chambers. Bacterial biofilms were allowed to develop under anaerobic condition as previously described (Yoon et al., [28]. After 24 hrs, biofilms were washed with sterile PBS to remove planktonic cells, and fresh LB broth (pH 6.5) containing 15 mM NO_3_ (control), or 15 mM NO_3_ plus 15 mM NO_2_, or 15 mM NO_3_ plus 15 mM NO_2_ and 10 mM c-PTIO, was added to the cultures. The biofilms were then incubated under anaerobic condition for an additional 48 hrs, washed 2 times with PBS and biofilm images were viewed by confocal scanning laser microscopy using an LSM 710 confocal microscope (Zeiss, Heidelberg, Germany) and visualized using a live (green cells)/dead (red cells) BacLight stain (Invitrogen, Eugene, OR). The excitation and emission wavelengths for green fluorescence (live cell) were 488 nm and 500 nm, while those for red fluorescence (dead cell) were 490 nm and 635 nm, respectively. All biofilm experiments were repeated at least 3 times independently. The dead/live ratios of the biofilms were calculated using imageJ 1.46r software following the guidelines by Christine Labno of the University of Chicago Integrated Light Microscopy Core. Briefly, the confocal image was imported into ImageJ, and the green (live cells) and red (dead cells) were separated resulting in only one color in each image. The color image was converted into a grey scale image. Then, adjust the threshold to highlight all of structures. At the end, the binary of the interested image was created. Finally, this image was used to analyze particles. Repeat the same cycle for the all other images. The percentage of dead/live ratio between the treated (NO_3_^−^ plus NO_2_^−^) and control (NO_3_^−^ only) was calculated and normalized.

### Effect of NO and KatA on anaerobic to aerobic growth transition

Wild-type strain *P. aeruginosa* PAO1 and its allelic mutants, Δ*katA*, Δ*nirS,* Δ*norCB,* Δ*katA*Δ*nirS* and Δ*katA*Δ*norCB*, were grown in LBN broth anaerobically for 24 hrs. The cultures were diluted in 10 ml of fresh LBN broth in a 250 ml flask and normalized to an OD_600_∼0.009. The cultures were then incubated aerobically with vigorous shaking and the cell density (as OD_600 nm_) recorded hourly.

### KatA overproduction and protein purification

The KatA-overexpressing bacteria BL21(DE3) (pLysS, pJFM18) were grown aerobically at 20°C to the OD_600_ = 1.0 in LB broth supplemented with 1 mM FeCl_3_. The culture was then treated with 1 mM isopropyl-β-D-thiogalactopyranoside (IPTG) and 2% ethanol for 10 hrs at 20°C. Bacteria were harvested by centrifugation at 10,000 × *g* for 10 min at 4°C, washed once with 0.9% saline, and the pellet was resuspended in a buffer containing 20 mM Tris-HCl-250 mM NaCl, pH 8.0. The bacterial cells were lysed by three passages through a French Pressure cell at 1,000 lbs/sq. in. The cell lysates were cleared by centrifugation at 13,800 × *g* for 30 min at 4°C and by filtration through 0.45 μm filter. Six-His-tagged KatA was first purified using His•Bind purification kit (Novagen, USA) following the manufacturer’s instruction, and then further purified using a GE Superdex 200 26/60 size exclusion column in 20 mM HEPES pH 7.0, 150 mM NaCl. The fractions representing tetrameric KatA were combined and concentrated to 5.5 mg/ml for crystallographic studies. The purity of recombinant KatA was assessed by 10% SDS-PAGE and staining with coomassie blue.

### Optical spectroscopy

Optical spectra were recorded on a GBC model 920 spectrophotometer (GBC, Australia). KatA was made anaerobic through repeated evacuation and exchange with argon on a Schlenk line. The concentrated enzyme was resuspended to ∼5 μM in a sealed cuvette with anaerobic buffer containing 50 mM Tris-HCl pH 8.0. Protein-NO bound complexes were formed by flowing NO gas over the sample for 1 min.

### Determination of the equilibrium binding constant (K_d_) of KatA for NO via spectrophometrically monitored ligand titration

Ferric KatA (as isolated) in 50 mM Tris buffer, pH 8.0, 250 mM NaCl was de-oxygenated under an argon atmosphere. The enzyme was transferred to a screw-cap style cuvette with a silicon septa lid. A saturated nitric oxide solution (1.9 mM, 23°C) was prepared by bubbling 6 M NaOH-scrubbed NO gas through Tris buffer. Each addition of the nitric oxide solution to the enzyme solution in the cuvette was made in an anaerobic glove bag. Optical spectra were recorded immediately after each addition on a Cary 50 Bio. NO was added until no further optical changes were observed. Absorbance increases at 574 nm were used to determine the fraction of NO-bound KatA. This value was plotted versus the amount of free NO (total NO added-KatA NO-bound fraction) and fit to a hyperbolic function to obtain the apparent *K*_d_ (Origin 8.6, Origin Lab, MA).

### Electron paramagnetic resonance (EPR) spectroscopy

#### (a) Purified KatA

100 μM KatA in the as-purified state in 50 mM Tris, 20% glycerol was frozen in liquid nitrogen. X-band EPR spectra were recorded using a Bruker Elexsys E-500 spectrometer equipped with an Oxford Instruments ESR-10 liquid helium cryostat, under the following conditions: Temp  =  2K, modulation amplitude  =  1 mT, and a microwave power of 100 μW.

#### (b) Intact bacteria

Different *P. aeruginosa* isogenic mutants were grown anaerobically in LBN or LBN, pH 6.5 for 24 hrs at 37°C and normalized to the same cell density. Suspensions were centrifuged at 10,000 x *g* for 10 min under anaerobic conditions and resuspended in 1∶10^th^ the volume of the original culture supernatant (∼0.5 ml). The concentrated suspension was added to SQ EPR tubes (Wilmad-Lab Glass, Vineland, NJ) in a Coy anaerobic chamber and capped with an air-tight plastic cap immediately before freezing the tubes in a dry ice-ethanol bath. DNIC EPR spectra were subsequently recorded on the X-band (9.33 GHz) Bruker Elexsys E-500 spectrometer with liquid nitrogen sample cooling (150 K), 10 G modulation amplitude, 1 mW microwave power.

### KatA crystallization and structure determination

Purified KatA was screened for crystallization conditions using an Art Robbins Phoenix crystallization robot. A single crystallization condition was obtained from the Hampton Research Index screen, which was optimized to the final conditions: 0.1 M sodium acetate, pH 4.3, 16% PEG 3350. Crystals of the approximate dimensions 50×50×100 μm were harvested and frozen in liquid nitrogen. Synchrotron data were collected at the Advanced Photon Source GMCA beamline 23ID, and a full dataset was obtained to 2.55Å. The structure of *Proteus mirabilis* catalase (Protein Data Bank accession 1M85) was chosen as a molecular replacement model due its high sequence conservation with *P. aeruginosa* KatA. Phaser identified four molecules in the asymmetric unit. The model was refined and manually rebuilt using PHENIX and COOT, respectively. Data collection and refinement statistics are shown in **Table 2**. The final structure was validated using MolProbity.

**Table 2:** KatA structure determination statistics.

**Data Collection**	
Space Group	P2_1_
Unit Cell	A = 67.73, B = 167.43, C = 90.55, α = 90.00, β = 111.39, γ = 90.00
Resolution (Å)	43.4–2.54 (2.57–2.54)
X-Ray Source	GMCA 23-ID-D
Wavelength (Å)	1.0332
Redundancy	4.1 (4.0)
Average I/σ(I)	15.58 (5.15)
Completeness (%)	98.63 (84.0)
R_sym_	0.086 (0.263)
Mosaicity	0.476
**Refinement**	
Reflections Used in Refinement	61322
Refined Residues	1922
Refined Waters	485
R_work_ /R_free_	17.57/20.05
Wilson B-value	25.23
Ramachandran plot§	
Favored (%)	97.12
Allowed (%)	2.88
Outlier (%)	0
R.M.S.D. Bonds (Å)	0.015
R.M.S.D. Angle	1.232
PDB ID Code	4E37

Figures in brackets indicate values for highest resolution shell.

R_free_  =  free R-factor based on random 5% of all data.

§ as calculated by molprobity.

### Detection of NO in bacteria

NO production by bacterial cells was measured using a fluorescence probe, 5,6 diaminofluorescein diacetate (DAF-2 DA) (Sigma-Aldrich, USA). Overnight aerobic cultures in LB broth were 1∶100 diluted into LBN in the presence of difference concentration of arabinose (0–0.1%), and grown anaerobically at 37°C for 24 hrs. One hundred microliter of bacterial cultures normalized to OD_600_ = 0.1 were incubated with 10 μM of DAF-2 DA in PBS for 30 min at 37°C. The fluorescent reaction product of DAF-2 and NO called DAF-2 T (excitation λ_max_  =  495 nm, emission λ_max_  =  515 nm) was measured with FL 600 microplate florescence reader, BIO-TEX. The relative NO level as measured by this fluorescent dye was calculated based on a calibration curve, which was generated by using varied concentration of MAHMA-NONOate, the NO donor and fluorescence signal output. The amount of DAF-2 T detected from each sample was then converted to a level of NO.

## Results

### PA catalase activity is increased under anaerobic conditions, specifically due to KatA expression, and not KatB or KatC

**Figure 1:**
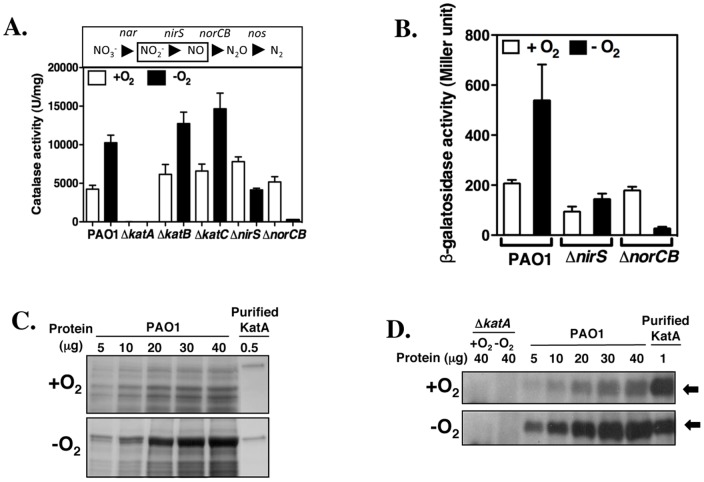
Analysis of catalase under aerobic and anaerobic conditions. A. Catalase activity of aerobic (white bars) vs. anaerobic (black bars) cell extracts of *P. aeruginosa* PAO1 and selected isogenic mutant bacteria. Bacteria were grown for 24 hrs in LBN broth under aerobic (+O_2_) or anaerobic (–O_2_) conditions. Cell extracts were assayed for catalase activity. The experiment was performed three times in triplicate. Standard deviation (SD) was shown. B. Analysis of *katA-lacZ* transcriptional activity under aerobic (white bars) vs. anaerobic (black bars) conditions in *P. aeruginosa* PAO1, Δ*nirS* and Δ*norCB* mutant bacteria. Bacteria harboring pQFp*katA* were grown for 24 hrs in LBN broth under aerobic (+O_2_) or anaerobic (–O_2_) conditions. Cell cultures were then assayed for β-galactosidase activity. The assay was repeated three times. SD was shown. C. SDS-PAGE and Coomassie blue staining of aerobic and anaerobic cell extracts of PAO1. Varied concentration of total soluble proteins (5, 10, 20, 30 and 40 μg) of either aerobic or anaerobic cell extracts of PAO1 were subjected to 10% SDS-PAGE electrophoresis and followed by Coomassie blue staining. Purified recombinant KatA (0.5 μg) was electrophoresed in parallel to indicate the migrating position of KatA on the gel. D. Western blot of aerobic vs. anaerobic cell extracts using anti-KatA antibody. The far right lane contains 1 μg of purified, recombinant KatA (black arrows). The number above each lane is the μg of cell extract loaded from aerobically or anaerobically grown bacteria. See Material and Methods for details.

We [15], [19], [52] previously showed that *PA* generates greater catalase activity during anaerobic versus aerobic respiration yet the molecular basis for this event and the anaerobic function of catalase remained unclear. Increased anaerobic catalase activity was found attributable to KatA since a Δ*katA* mutant possessed no catalase activity, while deletion of either KatB or KatC had no effect **(** **Fig. 1A** **)**. To elucidate the mechanism underlying the higher anaerobic KatA activity of wild-type strain PAO1, we first assessed the transcription of *katA* under aerobic and anaerobic conditions. A p*katA*-*lacZ* transcriptional fusion reporter plasmid, pQFp*katA*, was constructed and transformed into PAO1 via electroporation. β-galactosidase assays showed that anaerobic PAO1 harboring plasmid pQFp*katA* exhibited approximately 2.5-fold higher *lacZ* reporter activity than aerobic bacteria (**Fig. 1B**), indicating that transcription of *katA* is increased anaerobically, similar to catalase activity. Next, we examined the protein level of KatA from total soluble cell lysates of aerobic and anaerobic cultures. Both Coomassie blue staining (**Fig. 1C**) and Western blotting (**Fig. 1D**) clearly demonstrated that anaerobic cells produced significant higher KatA enzyme (∼2.5% of total soluble protein).

### Role of NO in KatA activity and *katA* gene expression

In order to address the possible role of metabolic NO in the observed increase in KatA activity of anaerobic PAO1, we examined catalase and p*katA*-*lacZ* reporter activities in an isogenic NIR mutant (Δ*nirS*) [42], a strain that is incapable of enzymatically-catalyzed NO production, under aerobic and anaerobic conditions. When compared to PAO1, the Δ*nirS* mutant yielded wild-type levels of aerobic catalase activity. However, when cultured anaerobically, the mutant produced much lower catalase activity, approximately half of that expressed aerobically and less than that of wild-type PAO1 (**Fig. 1A**), indicating that NO plays a role in the elevated anaerobic KatA activity of the wild-type PAO1. We also measured catalase and *pkatA-lacZ* reporter activity in an isogenic Δ*norCB* mutant, a strain that is deficient in NO reductase and accumulates higher levels of intracellular NO under anaerobic conditions [42]. As expected, the Δ*norCB* mutant produced wild-type levels of aerobic catalase activity. However, when cultured anaerobically, the mutant yielded very little catalase activity (**Fig. 1A**), which was purportedly inconsistent with the notion that higher KatA activity would be expected because of higher NO production in the Δ*norCB* mutant. Because this mutant is devoid of the ability to cope with such high levels of NO, this likely leads to the malfunctioning and/or paralysis of ANR (NO-sensitive)-mediated anaerobic respiratory machinery. This theory was corroborated by very poor anaerobic growth of the Δ*norCB* mutant and inactivation of recombinant ANR [42]. The reduced anaerobic catalase activity in both the Δ*nirS* and especially in the Δ*norCB* mutant was also reflected at the transcriptional level where we observed higher *katA-lacZ* activity in wild-type anaerobic cultures, little, if any, increase in reporter activity in the Δ*nirS* mutant, and extremely low transcription of the *katA* gene in Δ*norCB* mutant bacteria **(** **Fig. 1A and 1B** **).**

**Figure 2:**
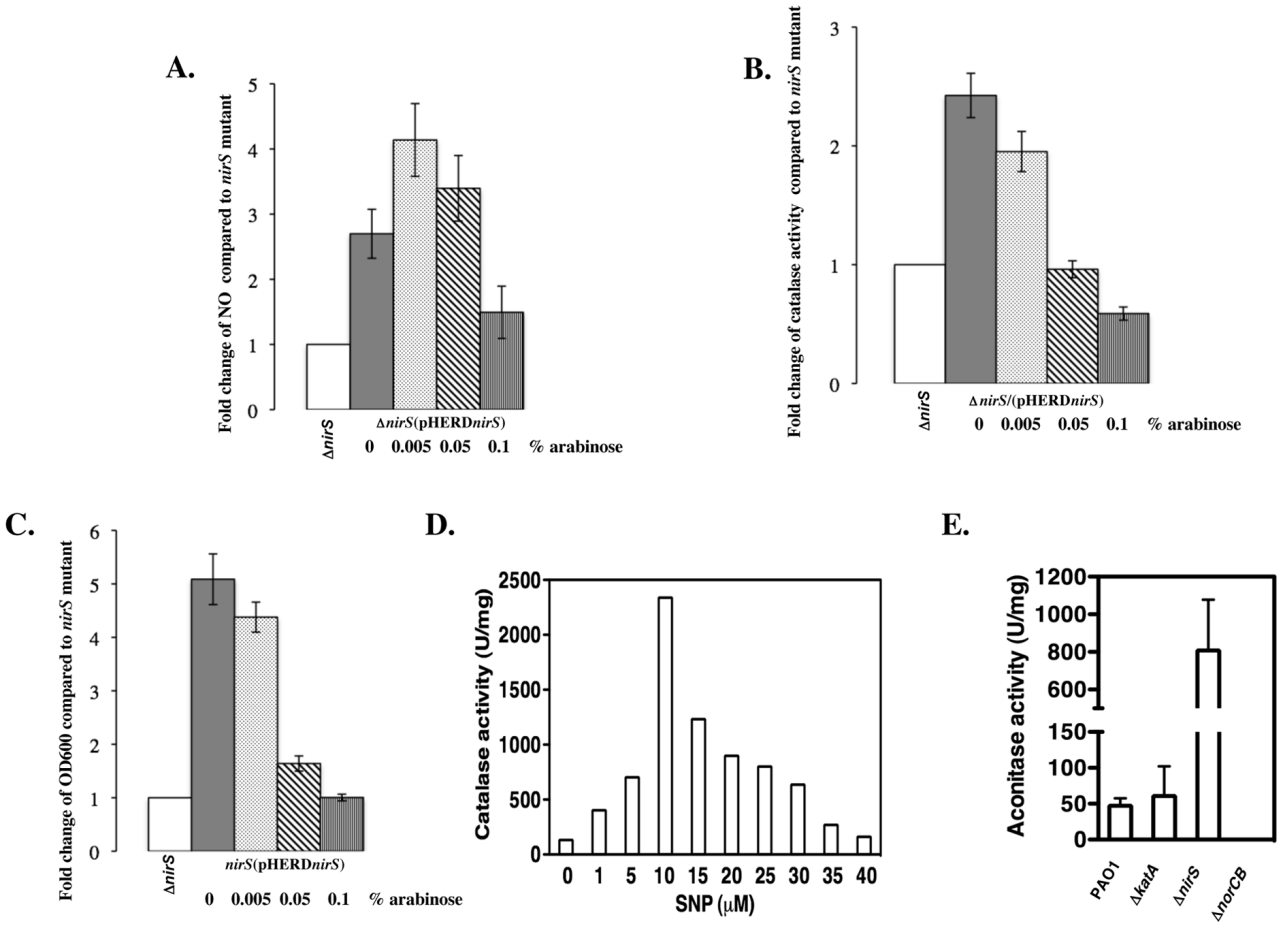
Effect of NO production on cell growth and catalase activity in Δ*nirS* mutant, the wild-type PAO1 and its isogenic mutants. *P. aeruginosa* Δ*nirS* mutant and Δ*nirS*(pHERD*nirS*) were cultured anaerobically in LBN for 24 hrs. Different concentration of arabinose (0–0.1%) were added to the cultures of strain Δ*nirS*(pHERD*nirS*) to induce expression of the *nirS* gene. Cells were sampled to measure NO production (A), catalase activity (B) and cell growth (OD_600_) (C). NO levels were determined using the fluorescent indicator compound, DAF-2A. Overnight anaerobic cultures were normalized and incubated with 10 μM of DAF-2A for 30 min at 37°C. Catalase activity was measured spectrophotometrically by following the decomposition of 19 mM H_2_O_2_. See Materials and Methods for detail. All results were presented as a value of fold changed compared to the Δ*nirS* mutant that was assigned a value of 1. The black, light gray and white bars represented PAO1, Δ*katA* mutant and Δ*nirS* mutant, respectively. The gray, dotted, diagonal striped and vertical striped bars were referred to Δ*nirS*(pHERD*nirS*) exposed to 0, 0.005, 0.05 and 0.1% arabinose, respectively. D. Catalase activity of the Δ*nirS* mutant upon SNP treatment. Exponential phase aerobic culture of Δ*nirS* in LBN was sub-cultured anaerobically with varying concentration of the NO donor SNP for 24 hrs. Cells were harvested, disrupted by sonication and the cleared cell lysates were used to measure catalase activity. Representative data are shown. E. Aconitase activity assay. Cells of anaerobically-grown PAO1, Δ*katA*, Δ*nirS* and Δ*norCB*. The assay was performed three times as described previously [51], and the standard deviation was shown.

Next, we elected to examine the effect of NO production on catalase activity and cell growth. We used a Δ*nirS* mutant harboring the plasmid, pHERD*nirS*, in which expression of *nirS* is inducible by arabinose. The low levels of NO detected in the Δ*nirS* mutant are likely a function of the reduction of NO_2_^−^ when NO_3_^−^ is not being utilized. When compared to the Δ*nirS* mutant, the Δ*nirS*(pHERD*nirS*) strain yielded ∼2.5 fold higher levels of NO (**Fig. 2A**) and catalase activity (**Fig. 2B**) and 5-fold greater cell growth (**Fig. 2C**), even in the absence of arabinose, indicating a leaky expression of *nirS* via the P_BAD_ promoter in *PA*. NO levels were increased after the addition of 0.005% arabinose (**Fig. 2A**), while no increase was observed in catalase activity and cell growth (**Fig. 2B,C**). However, the addition of 0.05% arabinose actually impaired cell growth (**Fig. 2C**) and also reduced catalase activity (**Fig. 2B**), likely due to harmful NO levels generated compared to the control (Δ*nirS* mutant bacteria). When arabinose concentrations were raised to 0.1%, NO production, catalase activity and cell growth of Δ*nirS*(pHERD*nirS*) dropped to levels similar to that of the Δ*nirS* mutant **(** **Fig. 2A,B,C** **)**. Exposure to an NO donor, sodium nitroprusside (SNP, 10–50 μM), to purified KatA *in vitro* did not significantly increase or decrease KatA activity (data not shown), in contrast with the results observed by Gusarov and Knudler using purified *Bacillus subtilis* catalase [53]. However, when SNP was added to live anaerobic Δ*nirS* mutant bacteria, catalase activity dramatically increased when exposed to 10 μM SNP (**Fig. 2D**), However, catalase activity decreased dramatically in Δ*nirS* bacteria when (i) *nirS* expression was elevated (**Fig. 2B**) and (ii) when such organisms were exposed to higher SNP concentrations than 10 μM (**Fig. 2D**), indicating that KatA expression was not sufficient enough to counter the amount of NO generated, and that excess NO was toxic to the bacteria, consistent with our previous results (Yoon et al., [42]).

One enzyme that is critical for normal cellular metabolism and very sensitive to NO is the [4Fe-4S]^2+^ cluster TCA cycle enzyme, aconitase [54]. We postulated that the reduction in KatA activity and cell growth upon exposure to elevated NO, as well as the significantly reduced (43-fold) anaerobic KatA activity in the Δ*norCB* mutant relative to wild-type cells (**Fig. 1A**) were attributed to the harmful level of NO reached by the cells and failure to detoxify NO. If this is the case, a reduction or loss of aconitase activity would be demonstrated in those cells. As expected, the Δ*norCB* mutant, that produces nearly 13.6 μM NO anaerobically, [42]) possessed no detectable aconitase activity (**Fig. 2E**). The Δ*nirS* mutant, that produced no enzymatically-driven NO, produced 16-fold higher aconitase activity than wild-type bacteria (**Fig. 2E**). Based on our previous studies [28], where Δ*rhlR* mutant bacteria were found to overproduce NO, they also possessed reduced aconitase activity. We hypothesize that NO-mediated inactivation of proteins such as ANR and aconitase, especially in the case of the Δ*norCB* mutant, is likely to be a result of NO-mediated destruction or modification (e.g., [2Fe-S] of the [4Fe-4S]^2+^ cluster of these proteins [42]). Such an event likely represents a last ditch, “circuit breaker” effort for the anaerobic Δ*norCB* mutant that is under considerable endogenous NO stress (13.6 μM) to retain cell viability, and indicative of abysmally slow growth patterns.

### Role of ANR in *katA* transcription and KatA activity

**Figure 3:**
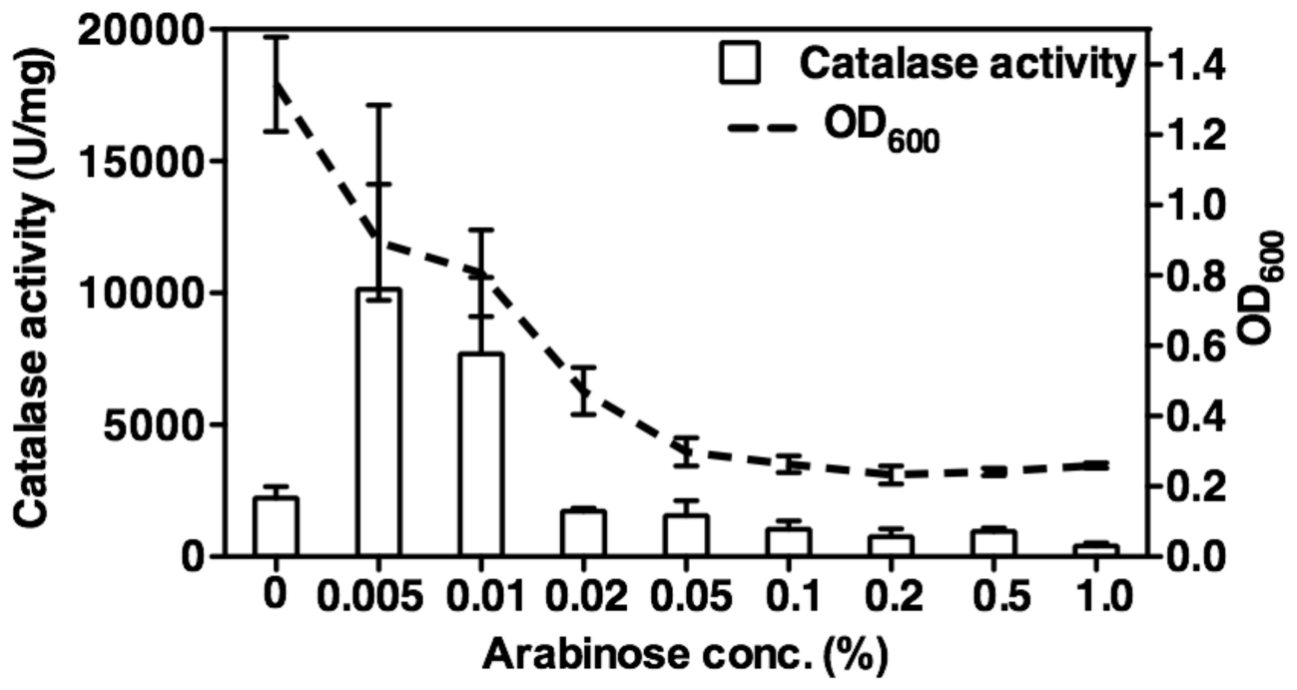
ANR upregulates the expression of the *katA* gene. The Δ*anr* mutant harboring plasmid pHERD*anr*, in which the expression of ANR is under the arabinose-inducible P_BAD_ promoter, was cultured anaerobically for 24 hrs in LBN supplemented with increasing concentration of arabinose up to 1%. Cultures were sampled for optical cell density (OD_600_) measurements (dotted line) and catalase activity (white bar). The experiment was performed three times independently and the standard deviation error bars are shown.

Trunk et al., [16] reported that the putative promoter region of *katA* has a weakly conserved ANR “box”, representing bases TTGTCTTCCGCCAA located 73-bp upstream of the translational start codon of *katA*. A slight Anr dependence of *katA* was detected in their transcriptome analysis and an Anr-dependent production of KatA was confirmed in their proteomic analysis under aerobic to anaerobic growth conditions [16]. To test that ANR participates in the regulation of the *katA* gene during anaerobic conditions, we first introduced plasmid pHERD*anr* into an Δ*anr* mutant that is incapable of anaerobic growth, in which the expression of ANR is under the control of the arabinose-inducible P_BAD_ promoter. The resultant strain, Δ*anr*(pHERD*anr*), was cultured anaerobically for 24 hrs in the presence of increasing arabinose levels, and both cellular growth (OD_600 nm_) and catalase activity were determined. As shown in **Fig. 3**, as little as 0.005% arabinose was sufficient and optimal to induce expression of catalase in the complemented Δ*anr* mutants via the induction of ANR expression. Increasing cellular levels of ANR beyond that point, however, decreased catalase activity and cell growth (**Fig. 3** **)**. This is likely due to the high iron and sulfur demands necessary for optimal ANR core Fe-S cluster formation and function, and also limiting these critical elements to serve other Fe-S cluster (estimated at >200 in *E. coli*, [32]) and other cellular proteins requiring these elements for normal function. Similar to the pHERD*nirS* plasmid (**Fig. 2A-C**), the expression plasmid pHERD*anr* was also leaky, manifested by cultures reaching their highest cell densities in the absence of arabinose. This is in marked contrast to the exceedingly tight arabinose control of the P_BAD_ system in *E. coli* [55].

### KatA helps protect planktonic PA against the potential therapeutic agent and NO donor acidified nitrite (A-NO_2_^−^)

**Figure 4:**
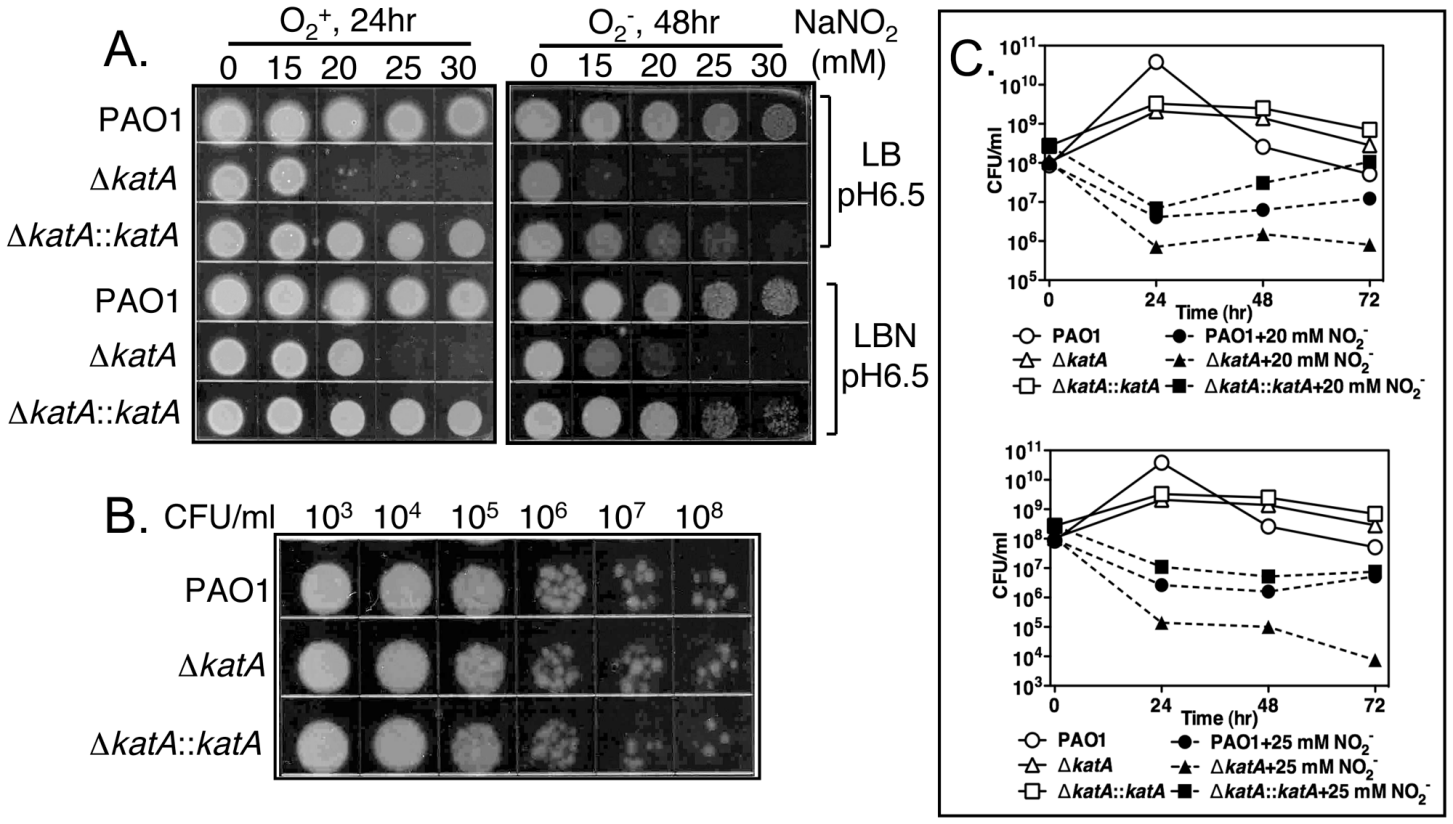
Sensitivity of the Δ*katA* mutant to A-NO_2_^−^. **A.** Wild-type strain *P. aeruginosa* PAO1, Δ*katA* mutant, and the complemented mutant Δ*katA*::*katA* were cultured aerobically in either LB broth (pH 6.5) or LBN broth (pH 6.5) for 24 hrs (**left panel**), or anaerobically in either LB broth (pH 6.5) or LBN broth (pH 6.5) for 48 hrs **(right panel)** supplemented with 0, 15, 20, 25 and 30 mM NaNO_2_, respectively. Cells were plated out directly on LB agar medium to determine the sensitivity to A-NO_2_^−^. **B.** Anaerobic cultures in LBN (pH 6.5) without A-NO_2_^−^ were serially diluted and spotted on LB agar plates to demonstrate the similar growth occurred in all test strains. **C.** Wild-type PAO1, Δ*katA* mutant, and the complemented mutant Δ*katA*::*katA* were cultured anaerobically for 72 hrs in LBN broth (pH 6.5) supplemented with 0, 20 mM, and 25 mM NaNO_2_, respectively. Culture aliquots were removed daily, and serial cell dilutions were plated out on LB agar medium to enumerate CFU. The experiments were repeated three times and the representative data were shown.

Our underlying hypothesis was that KatA plays a role in protection of anaerobic bacteria against metabolically generated NO, but especially against NO generated from outside the cell. An example of this is that during human infection, inducible NO synthases (iNOS) that generate NO contribute to the antimicrobial activity of macrophages and neutrophils [56]. Thus, we elected to use an NO donor, A-NO_2_^−^, that we have previously shown kills the antibiotic-resistant mucoid *mucA22* form of *PA* [40]. Both aerobically- and anaerobically-grown PAO1 Δ*katA* mutant bacteria exhibited greater sensitivity to increasing concentrations of A-NO_2_^−^ than wild-type bacteria or an *in cis* complemented Δ*katA* mutant (**Fig. 4A** **)**. Under both aerobic and anaerobic conditions, all A-NO_2_^−^-treated wild-type bacteria showed cell growth on plates, though decreased growth of anaerobic cells was observed when exposed to higher concentrations of A-NO_2_^−^ **(** **Fig. 4A, right panel** **).** In contrast, the Δ*katA* mutant bacteria exhibited varied sensitivities to A-NO_2_^−^, as manifested by poor or no growth on LB plates at specified exposure times and test conditions (i) 20 mM A-NO_2_^−^ or above in LB + O_2_; (ii) 25 mM A-NO_2_^−^ or above in LBN + O_2_; (iii) 15 mM A-NO_2_ or above in LB –O_2_; and (iv) 25 mM A-NO_2_^−^ or above in LBN –O_2_. As expected, the complemented Δ*katA* mutant, Δ*katA*::*katA*, with wild-type *katA in cis* at the chromosomal *attB* site were not susceptible to A-NO_2_^−^, similar to that of wild-type *PA*. As a control, wild-type PAO1, its Δ*katA* mutant, and the complemented mutant, Δ*katA*::*katA*, exhibited similar anaerobic cell growth in the absence of A-NO_2_^−^ where bacteria could grow only via NO_3_^−^ respiration (**Fig. 4B**). Since a single administration of A-NO_2_^−^ may not kill the entire Δ*katA* mutant population due to the presence of NIR activity, we performed viability (CFU) assays with bacteria exposed to 20 or 25 mM A-NO_2_^−^. In both cases that the Δ*katA* mutant was more sensitive to A-NO_2_^−^ than PAO1 and complemented Δ*katA* mutant organisms (**Fig. 4C**).

### KatA is important for protection of A-NO_2_^−^ sensitive mucoid, *mucA22* bacteria

**Figure 5:**
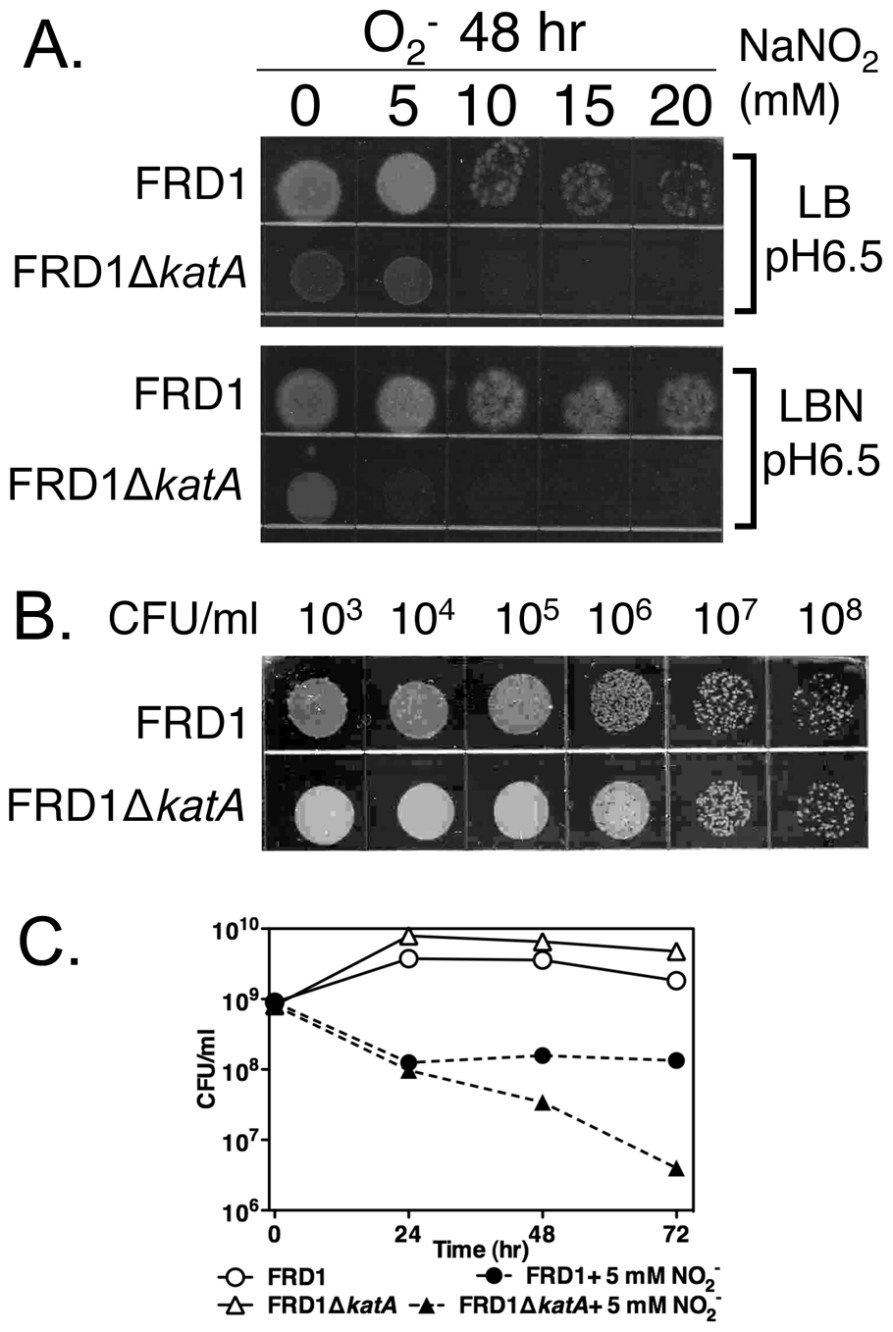
Sensitivity of the Δ*katA* mutant of mucoid *mucA22* strain FRD1 to A-NO_2_^−^. **A.** Undiluted samples of strains FRD1 and FRD1Δ*katA* that were subjected to various concentrations of anaerobic NO_2_^−^ treatment for 48 hrs were spotted on to LB agar plates. Top: samples anaerobically grown in LB, pH 6.5. Bottom: samples anaerobically grown in LBN, pH 6.5. **B** Anaerobic cultures in LBN (pH 6.5) without A-NO_2_^−^ were serially diluted and spotted on LB agar plates to demonstrate the similar growth occurred in test strains. **C.** Quantification of the bacterial load for each strain as measured by CFU/ml. Aliquots of anaerobic cell suspensions were removed from strains grown in LBN, pH 6.5 with the indicated concentration of A-NO_2_^−^. Serial dilutions were performed and the number of colonies were recorded after plating the dilutions on LB agar.

One hallmark of airway diseases such as COPD and CF is the frequent isolation of mucoid strains that have mutations within the *mucA* gene (notably *mucA22*) [57], [58] encoding a membrane-bound anti-sigma factor and the mutant was demonstrated to be sensitive to A-NO_2_^−^ [40]. Strain FRD1, a *mucA22* mutant coupled with a secondary *katA* mutation, renders such bacteria more sensitive to A-NO_2_^−^ than strain FRD1 alone (**Fig. 5A** **)**. **S**erially diluted control cells demonstrated identical CFU under anaerobic conditions without A-NO_2_^−^ (**Fig. 5B** **).** Notably, FRD1 Δ*katA* was sensitive to as little as 5 mM A-NO_2_^−^ relative to strain FRD1 alone (**Fig. 5C** **)**. This is in marked contrast to the higher levels (20-25 mM) of A-NO_2_^−^ required to kill nonmucoid PAO1-based strains (**Fig. 4**).

### KatA also helps protect anaerobic PA in biofilms upon exposure to A-NO_2_^−^

**Figure 6:**
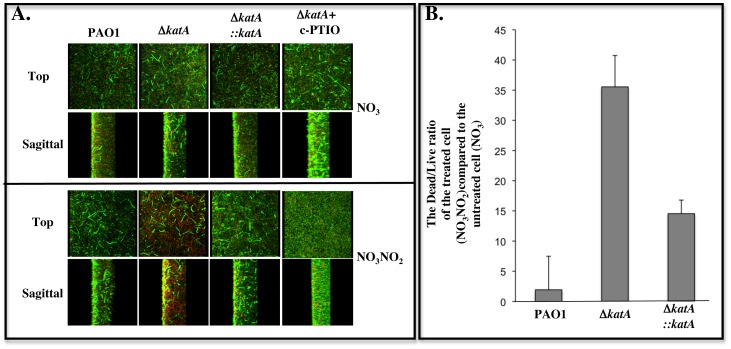
Susceptibility of Δ*katA* mutant biofilms to A-NO_2_^−^. Biofilms of wild-type *P. aeruginosa* PAO1, its Δ*katA* mutant, and the complemented mutant Δ*katA*::*katA* were grown anaerobically in LBN broth for 24 hrs. After gently washing off the planktonic cells, the biofilm cells were then cultured anaerobically in fresh LB, pH 6.5 with 15 mM KNO_3_, or 15 mM KNO_3_ + 15 mM NaNO_2_, or 15 mM KNO_3_ + 15 mM NaNO_2_ + 10 mM c-PTIO for additional 48 hrs. The biofilms were then stained with a viability stain containing the DNA binding agent Syto 9 (live, green cells) and dead (propidium iodide, red) according to the Materials and Methods, and such bacteria were observed by confocal laser scanning microscopy. **A.** Biofilm formation of *P. aeruginosa* PAO1, Δ*katA* mutant and Δ*katA::katA* in the absence of NaNO_2_ (top panel, top and sagittal views, serving as control biofilms) and in the presence of NaNO_2_ (lower panel, top and sagittal views, treated biofilms). Biofilms of the Δ*katA* mutant treated with 10 mM c-PTIO under both conditions were shown in far right panels. **B.** The ratio of dead/live cells in treated biofilms in **Fig. 6A** was calculated and normalized using ImageJ. The experiment was performed three times independently. The average values were plotted with standard error.

To test our hypothesis that KatA also plays a role in protection of anaerobic biofilm bacteria against A-NO_2_^−^, we treated mature anaerobic biofilm cells of PAO1 with A-NO_2_^−^. Strain PAO1 forms robust anaerobic biofilms compared to strain FRD1 [59] that has an LPS-rough phenotype [60] and lacks flagella [61], an appendage critical for biofilm formation [62]. The Δ*katA* mutant and the complemented mutant, Δ*katA*::*katA* was exposed to 15 mM A-NO_2_^−^ for 2 days, and viability assessed in such biofilms by live/dead staining of biofilm cells using confocal laser scanning microscopy. As shown in **Fig. 6A**, all three strains formed similar biofilms in LBN, pH 6.5, under anaerobic conditions **(top panel)**. In contrast, upon exposure to A-NO_2_^−^, Δ*katA* mutant bacteria exhibited greater levels of dead biofilm bacteria (**in red, bottom panel**) than wild-type *PA*. Genetic complementation by a chromosomal copy of wild-type *katA* at the non-essential *attB* site restored wild-type biofilm viability in the Δ*katA* mutant **(** **Fig. 6A** **, bottom panel)**. Upon chemical treatment with 10 mM c-PTIO, a well known NO scavenger [28], [42], biofilm cells of the Δ*katA* mutant exhibited the wild-type viability **(** **Fig. 6A** **, bottom panel)**. Based on the viability of untreated cells (**Fig. 6A** **)**, we further calculated and normalized the ratio of dead/live cells in A-NO_2_^−^ -treated biofilm. The treated Δ*katA* mutant biofilms harbored 14-fold more dead biofilm bacteria relative to wild-type bacteria (**Fig. 6B**).

### KatA and NO: Roles in anaerobic to aerobic growth transition

**Figure 7:**
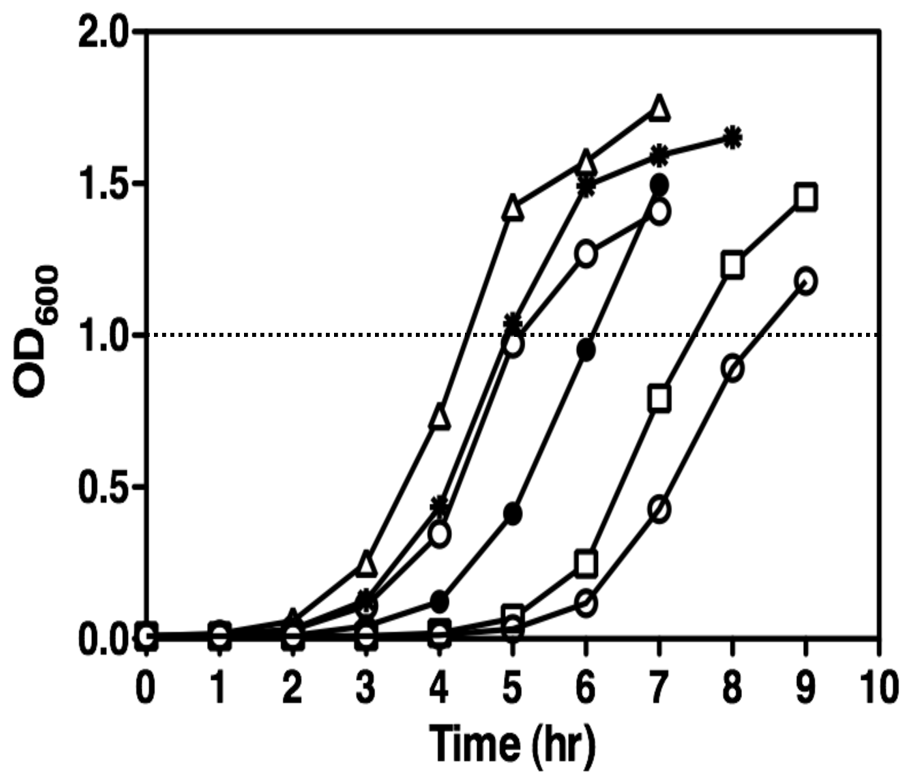
The importance of KatA in the transition from anaerobic to aerobic growth. Bacteria were grown anaerobically in LBN for 17 hrs. Cultures were diluted 1∶100 into fresh LB broth and incubated aerobically with vigorous shaking at 250 rpm at 37°C. The optical density was measured at hourly intervals. Open circles, PAO1; filled circles, Δ*katA*; open triangles, Δ*nirS*; open squares, Δ*norCB*; strikethrough circles, Δ*norCB*Δ*katA*; strikethrough asterisk; Δ*nirS*Δ*katA*. The experiments were repeated three times and the representative data were shown.

We next assessed the role of KatA in an anaerobic to an aerobic growth transition during which the H_2_O_2_ levels can suddenly elevate [63]. As shown in **Fig. 7**, the Δ*katA* mutant grew slower during the transition and took an extra hour to reach a similar rate of cell growth relative to wild-type *PA*, indicating that KatA activity was necessary for such a transition and may be one reason why anaerobic catalase is elevated, especially if organisms are suddenly exposed to molecular oxygen. Unexpectedly, the Δ*nirS* mutant grew more rapidly than even wild-type bacteria upon the transition from anaerobic to aerobic conditions, suggesting that an absence of endogenous NO (and possibly influenced by increased aconitase activity, **Fig. 2E**) enhanced the optimal transition from anaerobic growth to a more rapid aerobic growth rate. In striking contrast, the Δ*norCB* mutant, that is incapable of disposing anaerobic NO, grew much slower than even the Δ*katA* mutant during the transition. The mutant required two extra hours to reach wild-type growth rates and exhibited a much longer exponential growth phase. It was noteworthy that introduction of a Δ*katA* mutation in either Δ*nirS* mutant or Δ*norCB* mutant slowed cell growth, suggesting the importance of KatA during an anaerobic to aerobic growth transition, in which the growth rate of all strains in a simplified synopsis are as follows: Δ*nirS* > Δ*nirS*Δ*katA* > PAO1 > Δ*katA* >Δ*norCB* >Δ*norCB*Δ*katA* **(** **Fig. 7** **)**.

### Ferric KatA binds NO

With the knowledge in hand that KatA is critical for resistance to A-NO_2_^−^ in both planktonic and biofilm culture and important for an optimal growth transition from anaerobiosis to aerobiosis, recombinant KatA was purified to homogeneity to assess its potentially anaerobic NO-binding properties, a known property of catalases [64]. NO typically binds with much higher affinity to heme proteins in the ferrous rather than ferric state. However, attempts to reduce KatA to study the nitrosyl complexes were largely unsuccessful (data not shown). For example, we found that KatA was not reducible with typical reductants such as NADPH and dithionite, even in the presence of mediators such as methyl viologen. Photoreduction of KatA was attempted using deazaflavin and EDTA irradiated with visible light under anaerobic conditions as previously described for other catalases [65]. Even these conditions produced only partial reduction to the ferrous state (data not shown). Based on these observations, *in vivo* reduction as a means to form a strong NO complex seems unlikely, although, at present, this possibility cannot be ruled out.

**Figure 8:**
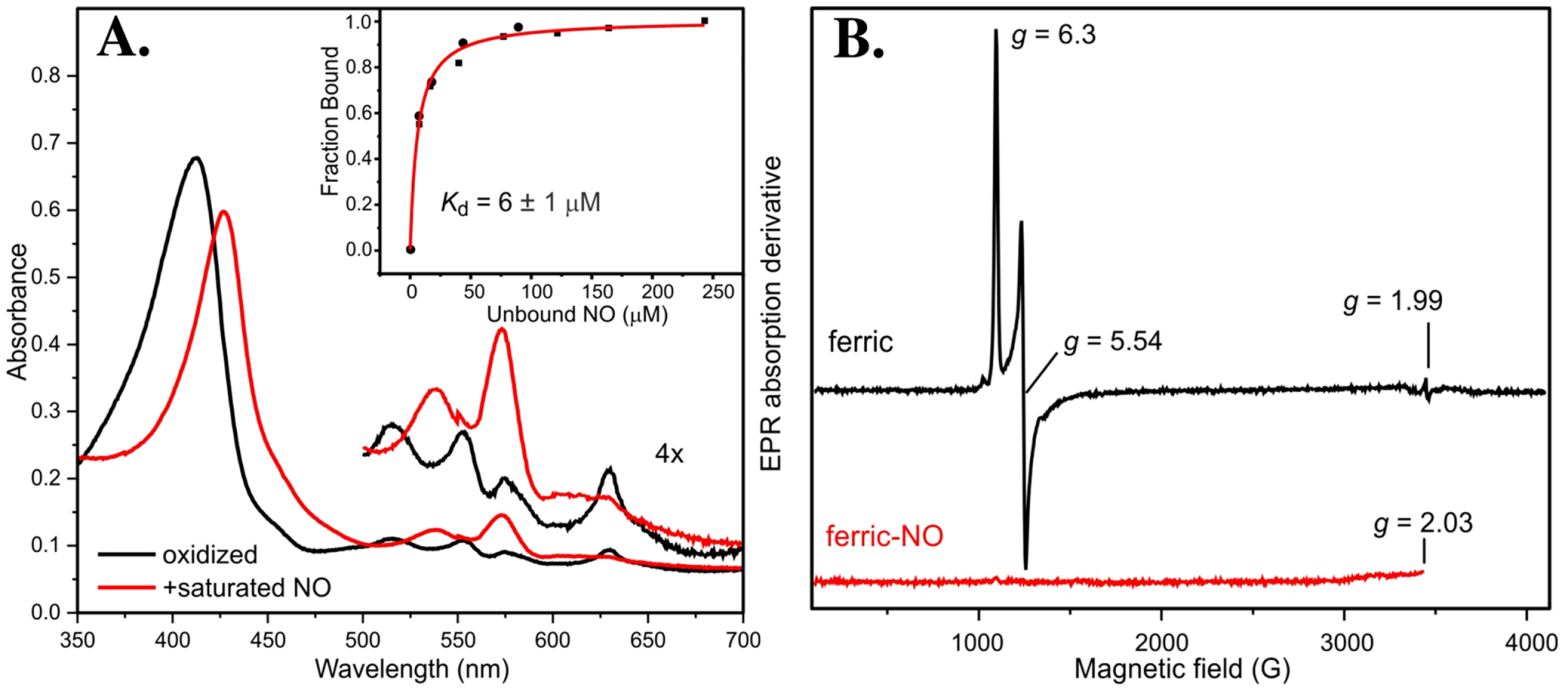
Effect of NO bound to oxidized KatA. **A.** Optical Spectrum of KatA in as-isolated state (black) and bound to NO (red**). Inset to part A:** **Titration curve of NO binding to KatA.** The titration was performed as described in Materials and Methods. Data from two independent titrations are shown superimposed (•,▪). The hyperbolic fit shown utilizes both data sets. **B. EPR spectrum of ferric KatA (black line) and NO-bound KatA (red line).** 100 μM KatA in the as-purified state in 50 mM Tris, 20% glycerol was frozen in liquid nitrogen. The spectral region below *g*  =  2.03 was obscured by the signal from dissolved NO. X-band EPR spectra were recorded using a Bruker Elexsys E-500 spectrometer equipped with an Oxford Instruments ESR-10 liquid helium cryostat, under the following conditions: Temp  =  2K, modulation amplitude  =  1 mT, and a microwave power of 100 μW. All experiments were performed three times and the representative spectrum was shown.

The optical Soret band of anaerobic ferric KatA is shown in **Fig. 8A** (black line). When NO was introduced into the system (**Fig. 8A**, red line), the spectrum of KatA immediately changed, exhibiting a Soret absorbance at 427 nm and Q bands at 540 and 574 nm, which are characteristic of NO binding to catalase [66]. At the same time, the charge transfer band at 630 nm typical of high spin ferric heme disappeared. Anaerobic titration of ferric KatA with NO showed that NO bound rather tightly to the heme iron, with an apparent *K*_d_ of 6 ± 1 μM **(** **Fig. 8A** **, inset**). These results are consistent with those found previously using bovine liver catalase [67]. After early fluctuations from 90 nM to nearly undetectable levels, the internal NO concentration for *PA* growing under anaerobic conditions is on the order of 0.41 μM ([42]); thus, significant complex formation *in vivo* seems likely.

The EPR spectrum at 2.2 K for KatA (**Fig. 8B** **, black spectrum**) shows that the metal center is homogeneous exhibiting *g*–values at 6.3, 5.54 and 1.99, characteristic of a high spin (*S*  =  5/2) ferric heme with a small distortion in axial symmetry. Generally, this is indicative of a 5-coordinate heme, which is consistent with the KatA crystal structure described below. This indicates that the heme iron in KatA is very likely to have an available binding site for small molecules such as NO. Paralleling the NO-mediated optical changes (**Fig. 8A**), the EPR spectrum of KatA was eliminated on NO binding (**Fig. 8B**), presumably due to spin coupling between the *S*  =  5/2 metal center and *S*  =  ½ NO. There are no new EPR-active NO-mediated KatA species generated or DNICs formed as evidenced by the absence of any new EPR signals down to g  =  2.03. Evaluation of the region at *g*  =  2 to determine if there is any ferrous KatA-NO formed was obscured by dissolved NO. However, the optical spectrum of the sample in the EPR tube shows no evidence for any species other than ferric KatA-NO (data not shown).

### KatA X-ray structure

The rapid and relative tight binding of NO to KatA as opposed to proteins without iron or heme suggests that a binding cavity large enough to accommodate NO and perhaps containing stabilizing moieties must be present. This was investigated by solving its crystal structure at a 2.55 Å resolution; the structural determination statistics are listed in **Table 2**. KatA is a class 3 catalase that forms a homotetramer (**Fig. S1A**). Each monomer of KatA binds a deeply buried heme group and an NADPH molecule at a peripheral solvent exposed site (**Fig. S1A and Fig. S1C**). The NADPH molecules co-purified with KatA during the protein purification steps, suggesting that KatA has a high affinity for NADPH. The proximal axial ligand of KatA is Tyr338 (**Fig. S1B**), the anionic charge of which may account in part for the difficulties encountered in reducing the enzyme. This charge would also be expected to weaken the affinity for NO, but this effect may be modulated by the interaction with nearby Arg334 as noted by Bandara et al., [66], for the NO complex of bovine catalase. The small molecule-binding pocket on the distal side of the KatA heme contains the conserved His (H55), which is essential for catalytic activity and its side chain forms a hydrogen bond with a nearby water molecule. Nearby, we modeled Met54 as a methionine sulfone, similar to what has been observed in the catalase of *Proteus mirabilis* [68]; the function of this sulfone modification has never been determined (**Fig. S1D**). The structure in the vicinity of the heme is very similar to that reported for bovine liver catalase. The NO complex of the latter enzyme has been structurally characterized [67]. Superposition of the two structures suggests that there would be no spatial constraints to NO binding in the KatA active site (**Fig. S1D**). Based on this alignment, KatA His55 and Phe141 and the active site solvent could potentially stabilize the binding of NO to the heme iron in the active site. One significant difference is that bovine liver catalase has no methione sulfone, but instead has a valine in its position ∼4 Å from the NO (**Fig. S2)**. If NO binds in the same orientation in KatA as it does in bovine catalase, then the Met-sulfone would be ∼5 Å from the NO, thus, far from hydrogen bonding distance. However, the sulfone side chain has considerable rotational freedom and could potentially assume a new conformation when NO is bound to KatA).

### KatA may modulate NO levels in anaerobic PA that lead to formation of dinitrosyl iron complexes (DNIC)

**Figure 9:**
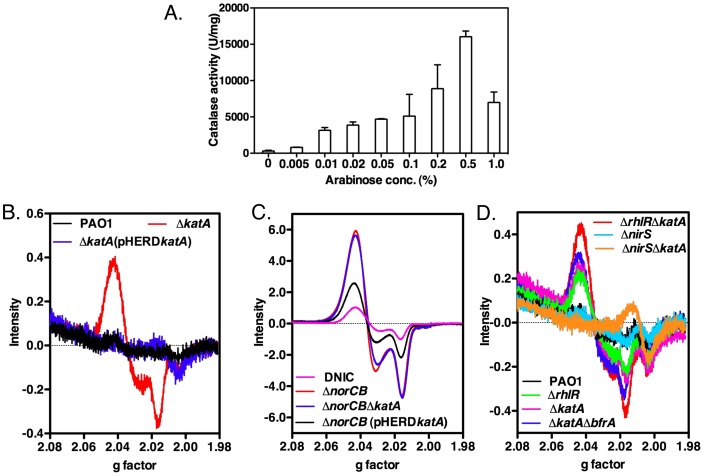
Whole cell EPR spectra of anaerobic bacteria. **A.** Catalase activity in Δ*katA*(pHERD*katA*) was induced maximally by 0.5% arabinose. Bacteria were grown anaerobically for 18 hrs in LBN broth supplemented with increased concentration of arabinose. Cells were harvested and lysed for catalase activity assay. EPR spectra were recorded on an X-band (9.33 GHz) Bruker Elexsys E-500 spectrometer at 10 G modulation amplitude, 1 mW microwave power at 150 K. **B.** Representative DNIC EPR spectra of anaerobic Δ*katA* mutant (red line) compared to overnight wild-type PAO1 cultures (black line) and Δ*katA* mutant (pHERD*katA*, blue line). **C.** EPR spectra of anaerobic cultures of Δ*norCB* (red line); Δ*norCB* Δ*katA* (blue line); and Δ*norCB* (pHERD*katA*, black line). **D.** DNIC EPR spectra of anaerobic cultures of Δ*nirS* (light blue line); Δ*nirS* Δ*katA* (brown line); PAO1 (black line); Δ*rhlR* (light green line); Δ*rhlR* Δ*katA* (red line), Δ*katA* (purple line); and Δ*katA* Δ*bfrA* (blue line).

DNIC formation in *PA* is a result of exposure of iron-containing proteins (e.g., Fe-S or Fe) to endogenously synthesized NO [69]. DNICs inherently have two NO molecules bound to an iron center. Fe-S metal centers are most often the sites of DNIC formation in *E. coli* [32]. A wide variety of such complexes may be produced, but they are all characterized by the appearance of an EPR resonance of *g*  =  2.04 [35]. DNICs have been proposed to have a variety of functions (i) act as signaling agents indicating NO stress [70], (ii) exert an “iron starvation” state in cells [71], (iii) compromise the function of proteins to which they are formed [32], or, more recently, (iv) proposed to facilitate repair of Fe-S clusters [72]. We hypothesize that *PA* KatA may help to decrease DNIC formation that could compromise the function of other iron-containing proteins, including ANR, the master regulator of anaerobic metabolism in *PA*. To test this hypothesis, we first measured catalase activity in a Δ*katA* mutant harboring plasmid pHERD*katA*, in which the expression of KatA was inducible by arabinose. As shown in **Fig. 9A**, KatA activity was induced gradually with increasing concentrations of arabinose, and reached the highest activity (17,000 U/mg) when exposed to 0.5% arabinose. Thus, we elected to use 0.5% arabinose for optimal expression of KatA and examined the effect of KatA on DNIC formation in *vivo*. Whole cell EPR experiments were performed with wild-type *PA*, Δ*katA* and other strategically selected mutants. The bacteria were grown anaerobically in LBN at pH 6.5, the approximate pH of the COPD and CF airways [40]. The frozen mutant suspensions normalized to total colony forming units were examined using liquid nitrogen EPR. **Fig. 9B** illustrated that the isogenic D*katA* mutant (red line) generates more DNIC (4.4-fold higher signal intensity at *g*  =  2.04) than wild-type bacteria (black line). Introduction of the plasmid, pHERD*katA*, into the D*katA* mutant reduced DNIC formation to the wild-type level (blue line). In contrast, **Fig. 9C** clearly showed the maximal formation of DNIC (63-fold higher signal intensity at *g*  =  2.04) at pH 6.5 in a D*norCB* mutant (red line) which we had shown accumulates micromolar levels (∼13.6 mM) of endogenous NO under anaerobic conditions [42]; RAW 264.7 (mouse macrophage cell line) cells grown in the presence of NO were used as a positive control for DNIC production **Fig. 9C**, purple line) [35]. We next postulated that deletion of the *katA* gene would be expected to increase the DNIC peak in a Δ*norCB*Δ*katA* double mutant (**Fig. 9C**, blue line). However, this was not observed in this strain due to the inherent high intensity of the signal coupled with the already extremely low KatA expression in D*norCB* mutant bacteria (recall **Fig. 1A**). Introduction of the *katA* gene *in trans* via the pHERD plasmid where the *katA* gene was overexpressed to levels shown in **Fig. 9A**, significantly reduced the DNIC signal by about 60% (**Fig. 9C**, black line).

Elimination of NO production by creation of Δ*nirS* or Δ*nirS* Δ*katA* mutants would be expected to result in less DNIC production as observed in **Fig. 9D** (light blue and brown lines). In contrast, anaerobic NO overproduction was induced using another mutant (Δ*rhlR*) devoid of the RhlR quorum sensing regulator (that we have shown overproduces NO during anaerobic growth [28]). **Fig. 9D** demonstrated that the Δ*rhlR* mutant bacteria (green line) produces substantial levels of DNIC, but the Δ*rhlR* Δ*katA* mutant strain (red line) increases this level by 50%. This shows, again, that the presence of active KatA attenuates DNIC formation by reduction of NO. We next tested a Δ*katA* Δ*bfrA* strain because we discovered that the *bfrA* gene, encoding an iron storage protein, BfrA, [10]), and potential NO binding protein, is immediately downstream of *katA* on the *PA* genome, but controlled by different promoters [10]. As expected, the DNIC spectrum in the Δ*katA* Δ*bfrA* mutant (blue line) is slightly greater than that of the Δ*katA* mutant (purple line). PAO1 (black line) also displayed little, if any, DNIC signal, presumably due to background KatA activity and normal NOR activity.

## Discussion

KatA is the major catalase of *PA*, the main function of which is to scavenge H_2_O_2_, a reactive oxygen intermediate produced spontaneously during aerobic respiration. The enzyme is one of the abundant proteins in *PA* (**Fig. 1C,D**) [10], is protease-resistant [17], and very stable [49]. Yet, we observed that anaerobic organisms possess higher catalase activity than aerobic organisms. From an energetics perspective, this seemed both wasteful and puzzling, since the substrate for KatA, H_2_O_2_, is not generated under anaerobic conditions. Prompted by this paradoxical increase in anaerobic catalase activity of *PA*, we hypothesized that KatA possesses other unknown function(s) or properties required by *PA* during anaerobiosis. First, we demonstrated that the elevated catalase activity was ascribed solely to the KatA, and not KatB and KatC, two other catalases of *PA* (**Fig. 1A**). Why is KatA activity increased under anaerobic conditions? In *Bacillus subtilis,* KatA was reported to be reactivated by NO directly, serving one of the mechanisms to counteract reactive oxygen species [53]. Hence, we postulated initially that the elevated catalase activity might be attributable to the reactivation of KatA by NO, an intermediate molecular generated by *PA* during the denitrification process. Unfortunately, we did not observe a boost in catalase activity in *PA* using either crude cell extracts or the purified KatA enzyme treated with NO (data not shown), indicating a behavioral difference of KatA from *PA* vs. that of *B. subtilis.* Examination of *pkatA-lacZ* transcription and KatA protein level revealed that anaerobic cells of PAO1 produced ∼2-fold higher reporter activity and a dramatically increased amount of enzyme than aerobic cells (**Fig. 1B, C and D**). Strikingly, the anaerobic KatA levels reached up to ∼2.5% of total soluble cell extract protein (**Fig. 1C,D**). Thus, we demonstrated that the increased transcription and expression of the *katA* accounted for the elevated catalase activity under anaerobic conditions. Based upon the experimental data gathered in this work, the previously unrecognized roles of elevated anaerobic KatA are discussed below.

### NO is required for optimal activation of anaerobic *katA* transcription and the master anaerobic regulator ANR controls this process

The surprising drop in anaerobic catalase activity in the Δ*nirS* mutant relative to wild-type *PA* suggested that NO is required for optimal transcription of the *katA* gene and/or enhances KatA protein stability (**Fig. 1A**). Clearly, the expression of *nirS* from a plasmid pHERD*nirS* and the exogenous addition of SNP resulted in increased NO levels and increased KatA activity in the Δ*nirS* mutant (**Fig. 2A,D**). Thus, at least basal levels of NO are required for high levels of KatA expression. This is likely due to the possible requirement of basal NO for optimal ANR transcriptional activity. Supportively, the [4Fe-4S] cluster of the *PA* ANR homolog in *E. coli*, FNR, reacts with eight NO molecules, resulting in oxidation of the cluster sulfide ions (S^2−^) to sulfane (S^0^). In fact, some NO molecules can remain associated with the protein as Cys persulfide [73]. This allows for FNR to act optimally as an anaerobic transcription factor, a mechanism that may also occur with *PA* ANR. Another alternative explanation is that reduced oxygen tension activates ANR, which, in turn, transcriptionally activates the *katA* gene as well as the NO-responsive second-tier regulator, DNR and other regulators for the denitrification (*nir*, *nor* and *nos* genes). Without NO, however, there is no transcriptional activation of DNR-dependent genes [74], leading to nonfunctional anaerobic respiration, resulting in poor growth of cells and less expression of KatA.

**Figure 10:**
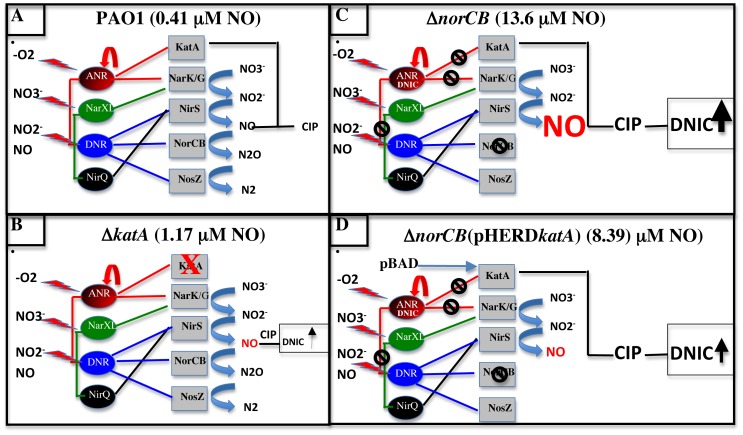
Model of anaerobic respiration in *PA* and the effects of KatA and NO. Depiction of the PA anaerobic anaerobic regulatory hierarchy and NO levels produced wild-type (**A**) (estimated baseline of 0.41 μM NO from Yoon et al., [42]), Δ*katA* mutant (**B**), Δ*norCB* mutant (**C**), and Δ*norCB* (pHERD*katA*) (**D**) strains. The central regulatory theme is based upon that described by Schreiber et al., [88]. The red “lightening bolts” indicate the nitrogen oxide species that control activation of specific anaerobic respiratory regulators (ANR (red spheres), DNR (blue spheres), NarXL (green spheres), NirQ (black spheres) with the exception of the *katA* gene. The colored lines emanating from ANR, DNR, NarXL and NirQ are those directed at downstream genes controlling the anaerobic respiratory cascade. The level of NO produced (in this case measured using an NO electrode or via extrapolation from DNIC levels in **Fig. 9**) and from Yoon et al., [42], in each organism is given on the top caption of parts A-D and also by a red color and font size, the size of which is directly correlative to levels of NO produced in each cell. The black circular “strike through” signs are assigned where genes are not activated. The arrow widths indicate the amount of DNIC produced in each mutant. The width of the black upraised arrows indicates the relative levels of DNIC (dinitrosyliron complexes) produced in each cell. CIP = chelatable iron pool.

Based upon the suggestion of Trunk et al., [16] that *katA* is under the control of ANR, we found that this event required production of minimal or basal NO levels. However, production of too much cellular NO or very high levels of ANR in the cell decreased catalase activity as well as cell density (**Figs. 2C,D** **and** **Fig. 3**). Similarly, very high NO levels dramatically reduced KatA activity in a Δ*norCB* mutant (**Fig. 1A**) and also eliminated NO-sensitive aconitase activity in this strain (**Fig. 2E**). Thus, when wild-type bacteria have low basal levels of NO, they produce more anaerobic than aerobic catalase activity (**refer to our model in** **Fig. 10**) and this process requires both the transcriptional activator ANR and at least a basal level of NO mediated by normal NIR activity.

### KatA is required for optimal anaerobic resistance to the NO generating agent, A-NO_2_^−^

In nature and during human airway diseases such as CF and COPD, bacteria often form antibiotic/biocide refractory, highly organized, enmeshed communities known as biofilms [28], [75], [76]. We have previously shown that KatA is essential for optimal viability of bacteria grown in aerobic flow-through biofilm systems when exposed to H_2_O_2_ [21]. In this study, we demonstrated that KatA was essential for optimal anaerobic viability of planktonic and biofilm bacteria and enhanced survival when cells were challenged with the potential therapeutic agent A-NO_2_^−^ in both the wild-type PAO1 and its formidable antibiotic-resistant, mucoid *mucA22* mutant (**Fig. 4** **–** **6**). The aforementioned mutant strains are frequently isolated from the lungs of both CF and COPD patients and severely complicate the clinical course for such patients. Like mucoid *mucA22* mutant of *PA*, *B. cepacia* has also recently been shown to be susceptible to killing by anaerobic A-NO_2_^−^ [77]. When oxygen is present, NO has been found to potentiate killing of *B. cepacia*, by formation of reactive oxygen intermediates (ROI) [78].

### Elevated anaerobic KatA is important for an optimal anaerobic to aerobic physiological transition

During biofilm culture and in some biofilm-related diseases (e.g., CF, COPD), there are macrocolonies harboring bacteria that are likely significantly deprived of oxygen [25] or even completely anaerobic [24], [28]. However, when bacteria become dislodged from the biofilm (often termed biofilm dispersion, [79]), they may be immediately exposed to ambient oxygen, leaving them vulnerable to a sudden and potentially lethal oxidative stress. Our data in **Fig. 7** indicate that KatA may facilitate the difficult transition from anaerobic to aerobic growth after suddenly being subjected H_2_O_2_ via aerobic respiration. In *E. coli*, fumarate reductase and the flavin-dependent desaturating dehydrogenase, NadB, are capable of generating H_2_O_2_ after a sudden anaerobic to aerobic transition [63], [59]. Intriguingly and unexpectedly, the Δ*nirS* mutant grew more rapidly when exposed to molecular oxygen than even wild-type bacteria (**Fig. 7**), indicating that the Δ*nirS* mutant cells have lesser cellular problems to deal with during the transition than other strains tested. In contrast, the Δ*norCB* mutant faced significant difficulties to grow up as manifested by the slowest growth (**Fig. 7**). Such differential bacterial growth during the transition was most likely attributable to the varied degree of NO-mediated cell damage or nitrosative stress occurred in anaerobic cells. Introduction of a Δ*katA* mutation in either Δ*nirS* mutant or Δ*norCB* mutant slowed cell growth, suggesting the importance of KatA during an anaerobic to aerobic growth transition.

### Anaerobic KatA likely acts as an internal buffer or a sensor to help initiate the detoxification process

Because NO is produced during anaerobic respiration by *PA* and many other denitrifying bacteria [80], and it has a high potential for interaction ­with iron, its levels must be stringently controlled by NO-detoxifying enzymes such as NorCB [31] and flavohemoglobin [81]. Our first hypothesis for elevated anaerobic KatA activity and its very high cellular protein levels is that it acts as a scavenger or “sponge” for NO at levels approaching its K*_d_* (e.g., ∼1 μM). The NO scavenging hypothesis for KatA, which crystallographically resembles the shape of a rectangular brick, with the long axis ∼100 Å and the short axis ∼50 Å, was initially derived from *in vitro* NO-KatA binding data, showing that the *K*_d_ for KatA with NO was relatively high, 6 ± 1 μM **(** **Fig. 8A** **, inset**). These results are consistent with those of bovine liver catalase [67]. The hypothesis was strongly supported by our *in vivo* EPR data obtained from several isogenic mutant strains. First, the Δ*katA* mutant generated 4.4-fold more DNIC than wild-type bacteria **(** **Fig. 9B**). Second, the overexpression of KatA from a plasmid not only restored the wild-type level of DNIC in the Δ*katA* mutant, but also reduced the culminating level of DNIC in Δ*norCB* mutant bacteria by ∼60% (**Fig. 9B,C**). Third, the level of DNIC in Δ*rhlR* mutant was increased by ∼50% with the introduction of *katA* mutation (**Fig. 9D**). The molecular basis for the protective effects of KatA on DNIC formation in *PA* may involve either buffering of free NO or a signaling event that may recruit other cellular defenses as toxic NO concentrations are approached. Hence, KatA catalase would serve to help protect the bacteria by buffering the NO, binding it when NO concentrations are elevated, but releasing it as concentrations fall so that NOR can degrade it. Such systems are likely overwhelmed in a Δ*katA* mutant, which demonstrated clear sensitivity to the NO generator, A-NO_2_^−^ in planktonic and biofilm bacteria (**Fig. 4** **,** **5** **and** **6**).

### KatA and similarities to anaerobic homogentisate-1,2-dioxygenase and hydroxyphenylpyruvate dioxygenase, non-obvious enzymes that bind to NO

Anaerobic KatA is similar in many respects to two oxygen-dependent proteins that are expressed in the anaerobic Δ*norCB* mutant, the iron-containing dioxygenases, homogentisate-1,2-dioxygenase (HmgA) and 4-hydroxyphenylpyruvate dioxygenase (Hpd) [42], respectively, that are typically only expressed aerobically. Initially, this finding seemed metabolically counterintuitive, since these latter enzymes require oxygen for enzymatic activity, yet their ANR-dependent, anaerobic de-repression (HmgA and Hpd overproduction) helped provide protection against impending cell death in biofilms because they help scavenge NO (supported by the EPR experiments with purified enzymes [42]), similar to experiments performed with KatA in this study. The NO produced in *norCB* mutant bacteria was high enough (∼13.6 μM) to impair the transcriptional activity of ANR via disruption of its [4Fe-4S]^2+^ cluster as shown by Mössbauer spectroscopy [42]. This, in turn, makes sense since ANR poisoning by NO in the Δ*norCB* mutant, enabling ANR-DNIC formation, would impair anaerobic *katA* transcription (**Fig. 1B**), resulting in significantly reduced KatA activity in this mutant (**Fig. 1A**) and resulted in a dramatic delay in the anaerobic to aerobic growth transition (**Fig. 7**). Similar to ANR and its homolog in *E. coli*, FNR, [82], NO also impairs other critical Fe-S proteins such as aconitase in *E. coli* [54] and the activity of this enzyme is essentially undetectable in a *PA* Δ*norCB* mutant (**Fig. 2E**). Fridovich and colleagues postulated that NO-mediated aconitase inactivation represented a “circuit breaker” like mechanism to prevent formation of additional reducing power in the form of NADH formed by the downstream TCA cycle enzyme, isocitrate dehydrogenase. In both ANR and aconitase, and likely many other Fe-S cellular proteins (>500 have been identified in different organisms, [83]), this compromised protein function is likely via formation of DNIC complexes. For example, Ren et al., [32] have observed, using anaerobic *E. coli,* more than 27 DNIC protein fractions with characteristic EPR *g*  =  2.04 signals ranging in molecular weight from >679 kDa to less than 29 kDa. Thus, while the identities of the proteins contributing to the very intense DNIC signal in anaerobic Δ*norCB PA* (**Fig. 9C**) remain unknown, we offer a model of how such events are influenced by KatA and NO in **Fig. 10A-D** **.** Again, this model is supported by the substantial decrease in the DNIC EPR signal when the *katA* gene is overexpressed in the *norCB* mutant in **Fig. 9C**. In wild-type anaerobic *PA*, NO levels rise to ∼90 nM within 15 minutes and then are reduced to nearly undetectable levels and yet eventually rise to ∼0.46 μM (based upon our DNIC conversions). This is due to the fact that ANR and its downstream regulator, DNR, which is activated by NO [84], are fully functional and activate the necessary NO detoxifying enzymes (e.g., NorCB) or proteins to which NO binds, Fe-S proteins (ANR, aconitase, KatA, HmgA, Hpd, *etc*.) that help the cell keep intracellular NO levels at very low levels (0.41 μM, **Fig. 10A**). **Fig. 10C** shows a schematic diagram of very high NO levels in the Δ*norCB* mutant with the chelatable iron pool (CIP) to generate very high DNIC levels, impair ANR, and thereby reduce anaerobic KatA activity by 43-fold (**Fig. 1A**). However, again, overexpression of KatA in the Δ*norCB* mutant bacteria, reduced DNIC production by ∼60% (**Fig. 9C**).

## Conclusions

In this study, we demonstrated that the elevated catalase activity in wild-type PAO1 under anaerobic conditions was attributed to the increased *katA* transcription and expression, which was controlled, in part, by the anaerobic response regulator ANR and required the basal level of NO. Compare to wild-type PAO1, anaerobically-grown planktonic and biofilm cells of the Δ*katA* mutant exhibited enhanced susceptibility to A-NO_2_^−^ and yielded higher levels of DNIC formation, which were restored to the wild-type levels by the complementation with the *katA* in *trans*. We further demonstrated a direct NO-KatA interaction *in vitro* using EPR, optical spectroscopy as well as X-ray crystallography. Collectively, our data strongly supported the conclusion that KatA helps protect *PA* cells against NO, an intermediate generated during anaerobic respiration. We proposed that KatA might be involved in buffering of free NO when potentially toxic concentrations of NO are approached within cells, but the basis for such protective effects exerted by KatA remains to be determined.

## Supporting Information

Figure S1 **X-ray structure of KatA.** **A.** **Ribbon diagram of the KatA tetramer.** Individual KatA monomers are colored yellow, magenta, green, and cyan. Each monomer binds a heme group, which is sequestered within the protein core, and a molecule of NADPH, which binds to a peripheral solvent exposed site. The atoms of the heme and NADPH molecules are represented as spheres with the carbons, oxygens, and nitrogens colored grey, red, and blue respectively. The tetramer has 222-point symmetry. **B.** **KatA active site.** Figure shows a close-up view of the KatA active site. The Cα backbone of a KatA monomer is shown and colored yellow. Catalytically important residues R52, M54, H55, R92, N128, R334, Y338, and R345 are in a stick representation with carbon, oxygen, and nitrogen atoms colored yellow, red, and blue, respectively. Due to compelling electron density, we modeled Met54 as a methionine sulfone, similar to what has been observed in other bacterial catalases [68]. The heme group is also in a stick representation with carbon, oxygen, and nitrogen atoms colored grey, red, and blue, respectively. The heme-bound iron is shown as a brick red colored sphere. **C.** **NADPH binding site.** Figure shows a close-up view of NADPH binding site. As stated earlier, four NADPH molecules co-purified with recombinant KatA during its purification, suggesting that KatA has a high affinity for NADPH. KatA residues are represented as sticks with carbon, oxygen, and nitrogen atoms colored yellow, red, and blue, respectively. NADPH is also in a stick representation with carbon, oxygen, and nitrogen atoms colored grey, red, and blue, respectively. KatA residues (R183, H193, H285, and L430), which interact with NADPH, are labeled. **D. Structural comparison of KatA with bovine liver catalase bound to NO.** The Cα atoms of KatA (PDB ID: 4E37) were overlayed with the structure of bovine liver catalase bound to NO (PDB ID: 3RGP). KatA is colored yellow and bovine liver catalase is colored grey. Residues involved in NO binding are labeled (KatA/bovine liver catalase). A water molecule that makes hydrogen-bonding interactions with the NO in the bovine liver catalase structure, potentially stabilizing the bound NO, is colored sky-blue. A corresponding water molecule was identified in the KatA structure, which makes hydrogen bonding interactions with H55, is colored cyan.
(TIF)

Figure S2 **Methione sulfone group in KatA structure.** KatA has a Met-sulfone group, ∼5 Å from the NO, while bovine liver catalase has a valine in the same position, ∼4 Å from the NO.
(TIF)
