# Supplementary figures and images for: Catalase (KatA) Plays a Role in Protection against Anaerobic Nitric Oxide in Pseudomonas aeruginosa
Source: PLoS One. 2014 Mar 24;9(3):e91813. doi: 10.1371/journal.pone.0091813 (PMC3963858; doi:10.1371/journal.pone.0091813)

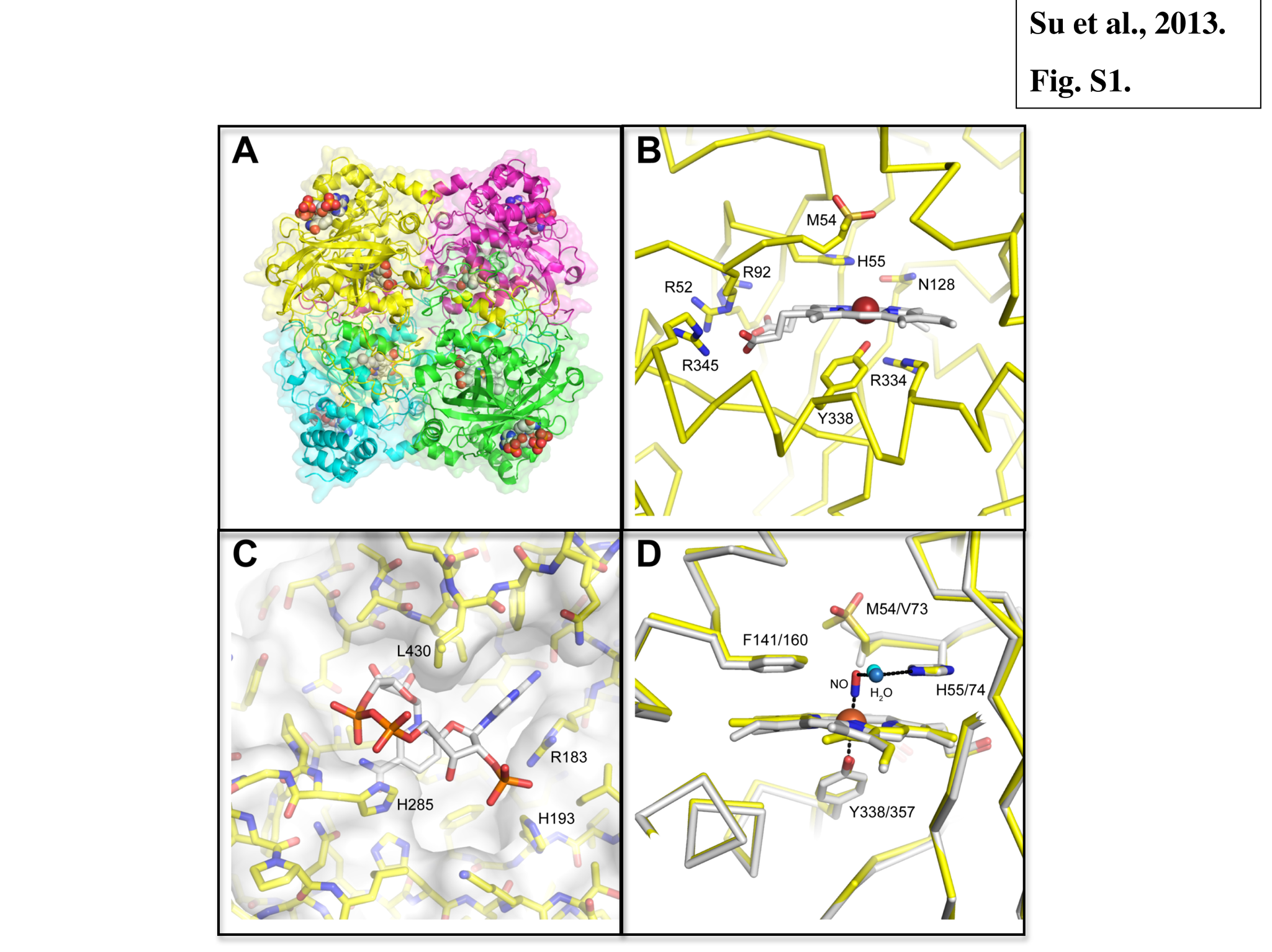

Supplement: Figure S1 — X-ray structure of KatA. A. Ribbon diagram of the KatA tetramer. Individual KatA monomers are colored yellow, magenta, green, and cyan. Each monomer binds a heme group, which is sequestered within the protein core, and a molecule of NADPH, which binds to a peripheral solvent exposed site. The atoms of the heme and NADPH molecules are represented as spheres with the carbons, oxygens, and nitrogens colored grey, red, and blue respectively. The tetramer has 222-point symmetry. B. KatA active site. Figure shows a close-up view of the KatA active site. The Cα backbone of a KatA monomer is shown and colored yellow. Catalytically important residues R52, M54, H55, R92, N128, R334, Y338, and R345 are in a stick representation with carbon, oxygen, and nitrogen atoms colored yellow, red, and blue, respectively. Due to compelling electron density, we modeled Met54 as a methionine sulfone, similar to what has been observed in other bacterial catalases [68]. The heme group is also in a stick representation with carbon, oxygen, and nitrogen atoms colored grey, red, and blue, respectively. The heme-bound iron is shown as a brick red colored sphere. C. NADPH binding site. Figure shows a close-up view of NADPH binding site. As stated earlier, four NADPH molecules co-purified with recombinant KatA during its purification, suggesting that KatA has a high affinity for NADPH. KatA residues are represented as sticks with carbon, oxygen, and nitrogen atoms colored yellow, red, and blue, respectively. NADPH is also in a stick representation with carbon, oxygen, and nitrogen atoms colored grey, red, and blue, respectively. KatA residues (R183, H193, H285, and L430), which interact with NADPH, are labeled. D. Structural comparison of KatA with bovine liver catalase bound to NO. The Cα atoms of KatA (PDB ID: 4E37) were overlayed with the structure of bovine liver catalase bound to NO (PDB ID: 3RGP). KatA is colored yellow and bovine liver catalase is colored grey. Residues involved in NO bindi [file pone.0091813.s001.tif]

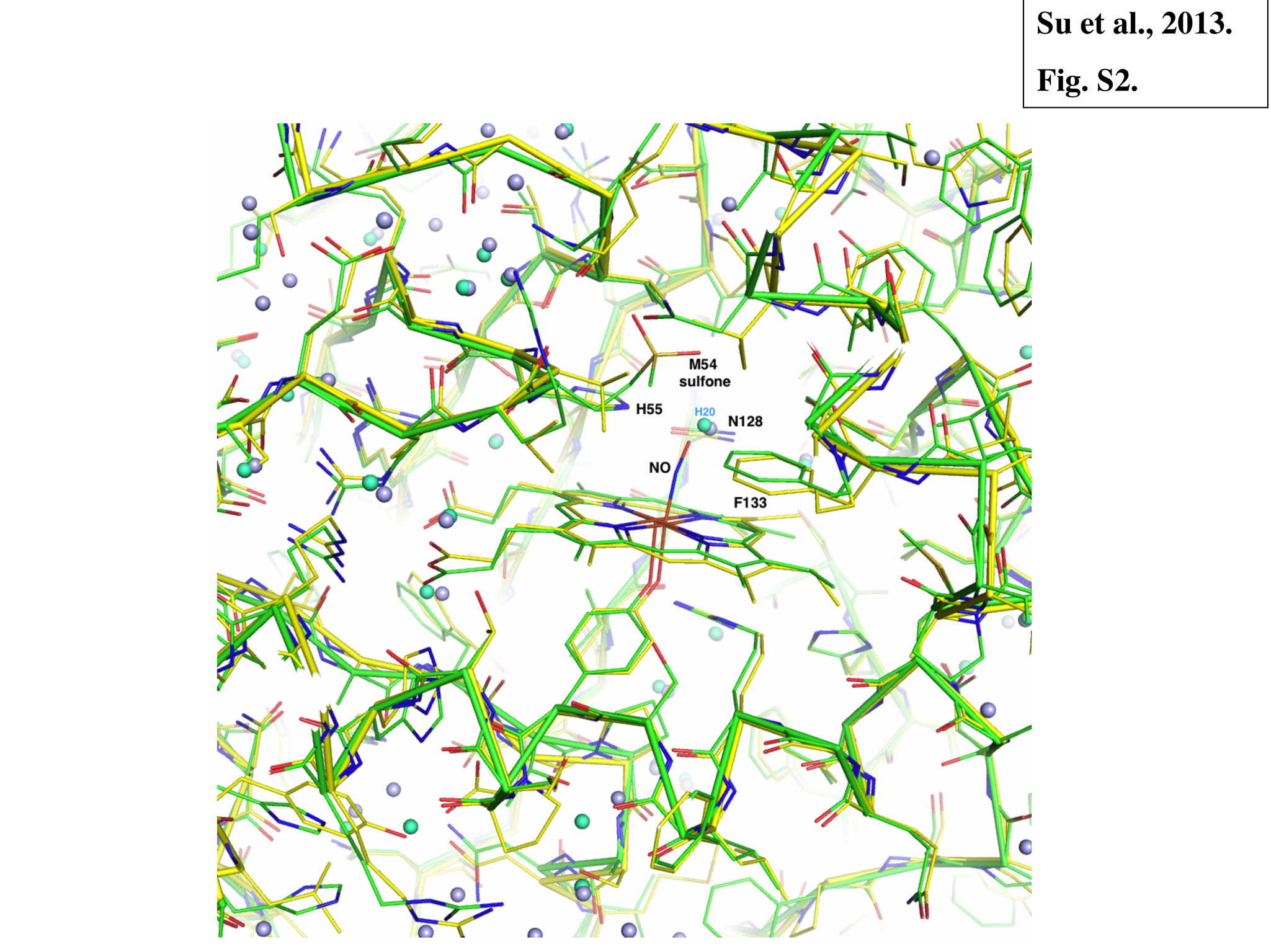

Supplement: Figure S2 — Methione sulfone group in KatA structure. KatA has a Met-sulfone group, ∼5 Å from the NO, while bovine liver catalase has a valine in the same position, ∼4 Å from the NO. (TIF) [file pone.0091813.s002.tif]
